# Paranormal believers show reduced resting EEG beta band oscillations and inhibitory control than skeptics

**DOI:** 10.1038/s41598-023-30457-7

**Published:** 2023-02-24

**Authors:** Abdolvahed Narmashiri, Javad Hatami, Reza Khosrowabadi, Ahmad Sohrabi

**Affiliations:** 1 School of Cognitive Sciences, Institute for Research in Fundamental Sciences (IPM), Tehran, Iran; 2 Bio-Intelligence Research Unit, Sharif Brain Center, Electrical Engineering Department, Sharif University of Technology, Tehran, Iran; 3 Shahid Beheshti University, Tehran, Iran; 4 University of Tehran, Tehran, Iran; 5 University of Kurdistan, Sanandaj, Iran

**Keywords:** Psychology, Human behaviour

## Abstract

Paranormal believers’ thinking is frequently biased by intuitive beliefs. Lack of inhibition of these tempting beliefs is considered a key element in paranormal believers’ thinking. However, the brain activity related to inhibitory control in paranormal believers is poorly understood. We examined EEG activities at resting state in alpha, beta, and gamma bands with inhibitory control in paranormal believers and skeptics. The present study shows that paranormal belief is related to the reduced power of the alpha, beta, and gamma frequency bands, and reduced inhibitory control. This study may contribute to understanding the differences between believers and skeptics in brain activity related to inhibitory control in paranormal believers.

## Introduction

Paranormal beliefs are beliefs, entities, practices, and processes that contradict the basic limiting laws of science^1^. Such beliefs include believing in traditional religions, telekinesis, superstition, witchcraft, spiritualism, magical thinking, extrasensory perception, and precognition^2^. Irwin^3^ hypothesizes that paranormal believers suffer from cognitive deficits. Supporting this hypothesis, some studies have shown deficiencies in the cognitive functions of paranormal believers. These deficits may include inhibitory control^4^, critical thinking^5^, probability misjudgment^6^, working memory and inattention blindness^7^, reasoning skills^8^, imagination^9^, cognitive and belief bias^10–12^. Human thinking is frequently biased; however, some people, such as paranormal believers, are more prone to biased thinking^13,14^. Cognitive and belief biases are mental errors that systematically lead to false tendencies, attitudes, and beliefs, and affect decision-making, reasoning, perception, and cognition^11,15^. Willard and Norenzayan^14^ stated that belief and cognitive biases, as byproducts of brain functions, form the paranormal experience’s biological basis. They suggest that supernatural agents appear from a set of correlated biases. Influenced by these biases, the human mind gravitates toward paranormal beliefs and intuitions. Intuitive beliefs often bias the thinking of paranormal believers^16^. Considering that several paranormal beliefs are built on statements that fail spectacularly when exposed to analytical consideration, one may anticipate an analytical thinking style to be adversely correlated with these beliefs^17^. Because the lower paranormal belief was correlated with higher analytical thinking^18^. However, concerning the reasoning interaction of interpretation inconsistencies, the dual-process theory in reasoning interaction is still under debate^19–21^. The main question is whether these two processes (intuitive vs. analytical thinking styles) compete to overcome each other, or whether a particular regulatory mechanism exists for these reasoning strategies. The mechanism that may be implicated as the suppressor of the intuitive thinking style is inhibitory control, which reduces the tendency toward paranormal beliefs^18^.

From a psychological perspective, the concept of controlling dominant biases and responses is related to the inhibitory control concept. Inhibitory control leads to the management of inappropriate or undesirable behaviors in humans, and it is considered one of the crucial components of executive functions^22^. According to Hood^23^, a paranormal sense may overwhelm some individuals who cannot adequately inhibit the rising intuitions. Consistent with this idea, poor inhibitory control is associated with teleological biases, and core knowledge confusion tendency^17^. Some studies have confirmed the inhibitory deficit in paranormal believers. Hood et al.^24^ showed that paranormal believers committed more errors than skeptics in the Wisconsin Card Sorting Test (WCST). Svedholm and Lindeman^17^ also showed that higher scores on ontological confusions and paranormal beliefs were correlated with poor inhibitory control in the Stroop task. In addition, Narmashiri et al.^25^ revealed that paranormal believers showed more errors in No-Go trials than skeptics in the Go/No-Go paradigm.

Electroencephalography (EEG) has been used to assess brain activity since more than a century. The EEG records the electrical activity of the brain across the scalp and provides a continuous index of electrocortical rhythms^26,27^. EEG is used in a variety of specific procedures. One popular method is known as resting-state EEG, which records continuing brain activity while the participant is not engaged in any specific cognitive task^28^. EEG activity during the resting-state, which is reliable over time, and reflects indices of brain activity, has been related to cognitive performance in previous studies^29–31^. Thus, evaluating the EEG resting-state activity may increase our knowledge about fundamental brain processes related to cognitive processes in paranormal beliefs. Event-related neural oscillations may be employed to analyze the neural responses through various frequency bands (e.g., delta, theta, alpha, beta, and gamma)^32^. Different cognitive functions are associated with each of these bands^33–36^. The alpha band activities are primarily related to cognitive control in the Go/No-Go paradigm^37^. In addition, several studies have been performed based on resting-state EEG focusing on inhibitory control in alpha, beta, and gamma band activities. For instance, Lee et al.^38^ stated that a reduction in alpha and beta band activities was associated with poor inhibitory control in the gambling disorder. Bresnahan and Barry^39^ revealed that ADHD individuals with high impulsivity had a lower activity level in the beta band. In addition, Choi et al.^40^ reported that reduced beta activity was associated with low inhibitory control in internet addiction. Moreover, there is a relationship between beta band activity, response preparation, and inhibitory control^41,42^, as well as cognitive function^43,44^. Studies have shown a weaker beta band power in No-Go trials in both non-human primates and healthy and neuropsychiatric human populations^42,45–49^. It is possible that increased inhibitory control results in increased beta band activity, while decreased inhibitory control results in reduced beta band activity^50,51^. According to these studies, beta power oscillations may be associated with inhibitory control. On the other hand, gamma power oscillations are associated with diverse cognitive functions from conscious awareness to inhibitory response^52–55^. Few studies have shown low gamma band activity in the No-Go trials related to inhibitory control^56,57^. Barry et al.^58^ suggest that ADHD children with high impulsivity have decreased beta and gamma absolute power. Benasich et al.^59^ showed a relationship between better cognitive functions, such as inhibition control, and an increased frontal gamma band activity in children with ADHD. In addition, low gamma power oscillations in the frontal lobe may indicate poor inhibitory control^60^.

Neuroimaging studies have linked inhibitory control to the right inferior frontal gyrus (IFG)^61,62^. However, there is little evidence of the underlying neural changes related to inhibitory control in paranormal beliefs. Nonetheless, Lindeman et al.^63^ showed that the rIFG area had less activation in paranormal believers based on functional magnetic resonance imaging (fMRI). In contrast, this area had strong activity in skeptics, indicating that less activation in the rIFG may be related to poor inhibitory control in paranormal believers. On the other hand, while a few studies have investigated resting-state EEG in paranormal believers^64–67^, the relationship between brain activity and specific cognitive processes, such as inhibitory control, has not yet been investigated, and data regarding the role of frontal lobe activity are lacking. Hence, Fleck et al.^68^ showed that individuals with high transliminality demonstrated low alpha, beta, and gamma band activities over the left parietal/occipital lobe, the right superior temporal cortex, and the left/right temporal lobe, respectively, compared to individuals with low transliminality. Additionally, Nash et al.^69^ found that religious believers showed increased dominance of a network related to intuitive processing. In contrast, non-believers showed increased power of a network linked to analytic processing. Furthermore, Kleinert et al.^70^ found that self-control, behavior related to self-control (i.e., risk-taking), and a neural measure of self-control were associated with longer durations of mental processes as identified in resting-state EEG recordings.

It seems that there is an association between the alpha, beta, and gamma oscillations and inhibitory control in the frontal lobe^57,60,71–73^. Previous studies in poor inhibitory control have shown reduced alpha, beta, and gamma oscillations in EEG under resting conditions. So far, no study has examined EEG activities during resting-state concerning inhibitory control in paranormal believers, who would be expected to show low levels of inhibitory control. Following the cognitive deficits hypothesis in paranormal believers^3^, we investigated resting-state EEG oscillatory activity to identify brain activation related to paranormal beliefs and inhibitory control. Since there aren't any EEG resting-state studies related to response inhibitory processes, inhibitory deficit, and reports of frontal lobe dysfunction in paranormal believers^4,25,74,75^, we predicted that paranormal believers would be characterized by reduced EEG oscillatory activity related to inhibitory control in the whole brain compared to skeptics. Paranormal believers are most likely to show poor inhibitory control compared to skeptics, which, as mentioned, may be related to beta band activity in the brain. Therefore, we hypothesize that paranormal believers are most likely to show poor performance in No-Go trials compared to skeptics. Moreover, we predict that paranormal believers show lower beta band activation across the whole brain. Additionally, frontal lobe is separately analyzed as it is associated with inhibitory control (which is related to paranormal belief) in the present study.

## Methods

### Participants

A total of 20 (10 female, 10 male; mean age = 22.50 years, SD = 4.07, age range 19–34) healthy right-handed (Edinburgh Handedness Inventory) students were selected for the study. We were looking for students who believed in or were skeptical about paranormal beliefs. The participants received gifts for participating in the present study.

Initially, 33 students were selected for this study. The severity of paranormal beliefs was determined using the overall score on the Revised Paranormal Belief Scale^76^. The distribution of paranormal beliefs was strongly skewed, as expected. Those belonging to the upper and lower groups (30%) were included in the analyses, i.e., paranormal believers (5 female, 5 male; mean age = 22.30 years, SD = 3.94, age range 19–27), and skeptics (5 female, 5 male; mean age = 22.70 years, SD = 4.39, age range 19–34). Based on previous studies^77,78^, we focused on the more extreme groups.

The participants' mean (SD) paranormal belief scores in the paranormal believer and skeptic groups were 84.80 (17.03) and 35.30 (6.23), respectively. According to the self-report questionnaire, the participants had no history of psychosis, mental illnesses, acute or chronic diseases, neurological or personality problems, alcohol or drug abuse, or epilepsy. This study was conducted following the principles of the Declaration of Helsinki, and approved by the Ethics Committee of The Institute for Cognitive Science Studies. All participants provided written informed consent before enrollment.

### Materials

The current study uses the Revised Paranormal Beliefs Scale (RPBS), which is used widely to assess belief in the paranormal. The RPBS consists of 26 items grouped in seven subscales, i.e., Traditional Religious Beliefs, Psi, Witchcraft, Superstition, Spiritualism, Extraordinary Life Forms, and Precognition. The items are rated on a seven-point Likert scale (from 1 = Strongly Disagree to 7 = Strongly Agree)^76^. Items are presented as phrases (e.g., "If you break a mirror, you will have bad luck"), and respondents evaluate the answers on a seven-point Likert scale according to how strongly they agree with them. The RPBS can give scores that corresponds to the subscales, and the aggregate yields a general estimate of the degree of paranormal belief. The RPBS subscales and the measurement show sufficient validity and reliability^76,79^. Internal consistency in previous study was excellent, with Chronbach's α = 0.92^80^.

The Cognitive Failures Questionnaire (CFQ) is a self-report questionnaire measuring failures in memory, perception, and motion in daily life. The CFQ consists of 25 questions, and the participants answer the items on a five-point Likert scale (from 0 = never to 4 = always). "Do you neglect to listen to people's names while you are meeting them?" is an example of a question. A high score shows a greater propensity for cognitive failure^81^. It has been demonstrated that CFQ has adequate validity and reliability, including high internal consistency and test–retest stability, and multidimensional factor structure^81–84^.

**Figure 1:**
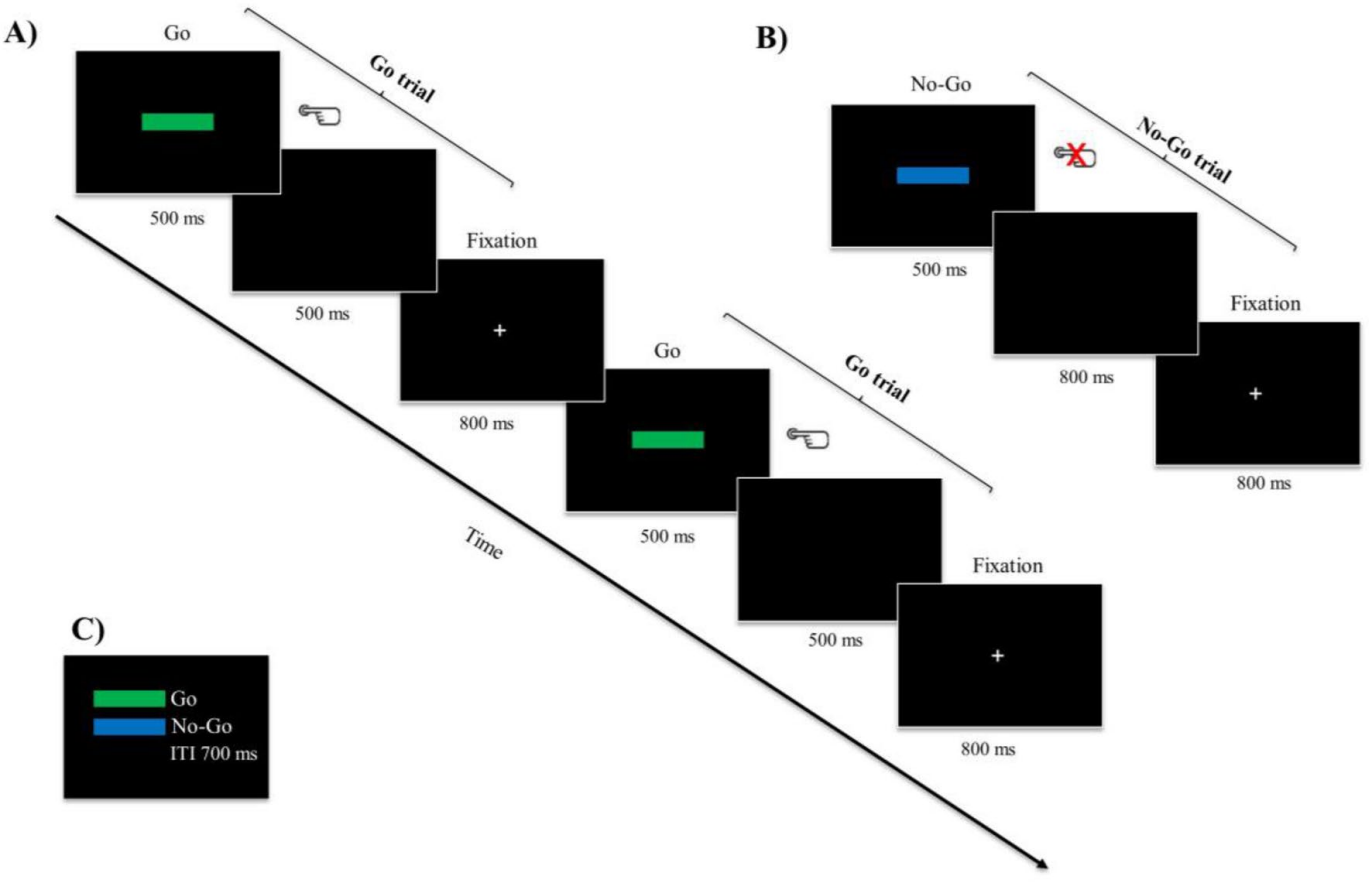
Go/No-Go task. (**A**) The procedure for go and (**B**) no-go trials was identical, apart from the instruction to press spacebar after the presentation of go stimuli and to withhold a response after no-go stimuli. After 500 ms of stimuli, a blank screen appeared for 500 ms. A fixation point was shown for 800 ms. (**C**) Illustrations of go- and no go stimuli. An inter-trial interval (ITI) of 700 ms was used. The Go/No-Go task was administered with Inquisit 5 (Millisecond Software, LLC).

The Go/No-Go Task is a behavioral measure of inhibitory control^85^. On a computer monitor, this task was presented. A fixation stimulus in the monitor's center always appeared before the visual Go and No-Go stimuli. The participants were instructed to press a button as rapidly as possible in response to the green rectangle that appeared in the center, which served as the go stimulus. The participants were asked to withhold their response to the No-Go stimulus (blue rectangle), which appeared in the center. The mean of errors (errors in Go trials, errors in No-Go trials, and error rate) were calculated based on the task performance. The Go and No-Go stimuli were presented in random (for details, see Fig. 1).

### Procedure

There were two stages to this experiment. First, participants completed the questionnaires including the demographic questions, RPBS, and CFQ. Second, participants (*N* = 20) were organized for an in laboratory component. The experiment was carried out by participants at a computer station within an electrically isolated EEG-cabin. They were fitted with a 32-channel EEG (Mitsar Medical, Petersburg, Russia) headset after initially providing written informed permission. Although there seems to be a difference between the eyes-open and eyes-close conditions^86^, there are still inconsistent results^87^. We used an eyes-open resting-state instead of an eye-closed resting-state, because the EEG recording was conducted in the early morning for all the participants. Participants were instructed to keep their eyes open while the EEG was being recorded to avoid drowsiness artifacts in line with previous studies^88–93^. Additionally, the eyes-open resting-state represents a more appropriate baseline for comparing two groups together^90,92^. EEG data were collected while participants stared at a blank screen for five minutes. Then, participants completed Go/No-Go task. Eventually, participants completed the manipulation and compliance checks. After receiving a debriefing, the headset was taken off, participants had their hair cleaned, and they were thanked for their participation.

### EEG recording, preprocessing, and analyses

The participants were seated in a resting state in a recording room. EEG recordings lasted for 5 min with eyes open. EEG recordings and acquisitions were made with a 32-channel MITSAR 202 system (Mitsar Medical, Petersburg, Russia). The standard 10–20 montage system was used, including FP1, FPZ, FP2, F7, F3, Fz, F4, F8, FT7, FC3, FCZ, FC4, FT8, T3, C3, CZ, C4, T4, TP7, CP3, CPZ, CP4, TP8, T5, P3, Pz, P4, T6, O1, Oz, and O2 electrodes. As a reference, a single channel with bipolar electrodes was attached to the ears. The location of the ground electrode was between FPz and Fz. The signal sampling was done at a frequency of 250 Hz. The electrode’s impedance was below 5 kΩ, and the EEG signals were band-pass filtered offline from 0.1 to 40 Hz. The WinEEG system’s recordings were transferred to the EEGLAB toolbox (NG 2.5.5; Applied Neuroscience, Inc., St. Petersburg, USA) for the standard preprocessing stage. Artifact removal was performed offline using the artifact rejection toolbox of the EEGLAB toolbox. EEG recordings were also visually and manually inspected to eliminate the effects of eye, muscle, movements, and other artifacts in line with previous studies^94–97^. Subsequently, spectral analysis was performed using a fast Fourier transform in MATLAB (MathWorks, Natick, Massachusetts, USA). Absolute (µV2) power was extracted from the cleaned EEG data in nine frequency bands, including delta (1–4 Hz), theta (4–8 Hz), alpha (8–13 Hz), alpha1 (8–10), alpha2 (11–13), beta (13–30 Hz), beta1(13–21), beta2 (19–30), and gamma (30–40 Hz). Following a previous study^75^, the activity at 31 sites was divided into four regions, two hemispheres, and eight hemispheres/regions by averaging within each region, hemisphere, and hemisphere/region. The four regions include frontal (FP1, FPZ, FP2, F7, F3, Fz, F4, F8, FT7, FC3, FCz, FC4, and FT8), temporal (T3, T4, TP7, TP8, T5, and T6), parietal (CP3, CP4, CPz, Pz, P3, and P4), and occipital (O1, O2, and Oz). The two hemispheres are the right (FP2, F4, F8, FC4, FT8, C4, T4, TP8, T6, CP4, P4, and O2) and the left (FP1, F3, F7, FT7, FC3, C3, T3, TP7, T5, CP3, P3, and O1). In addition, the eight hemispheres/regions are left frontal (FP1, F3, F7, FT7, and FC3), right frontal (FP2, F4, F8, FC4, and FT8), left temporal (T3, TP7, and T5), right temporal (T4, TP8, and T6), left parietal (CP3 and P3), right parietal (CP4 and P4), left occipital (O1), and right occipital (O2).

### Statistical analysis

Comparisons of demographic (age, gender, and education) and behavioral variables (PRBS scores, CFQ scores, error rate, error in Go and error in No-Go trials in Go-No/Go task) between the groups (i.e., paranormal believers and skeptics) were conducted using the independent two-sample t-test and chi-square tests. EEG absolute power data were analyzed separately for all the bands, the areas, the hemispheres, and the hemispheres/regions using an independent two-sample t-test between two groups (paranormal believers vs. skeptics) with adjusted p-values using the false discovery rate (FDR) method to limit type I error^98^. FDR correction was not performed in correlation results due to the exploratory nature of this stage. The effect size for the t-test was calculated using Cohen's d. In addition, we used Pearson’s correlations to explore the relationships between resting-state EEG activities and demographic/behavioral variables in paranormal believers and skeptics. A multiple regression analysis was performed to show the effect of frequency bands (delta, theta, alpha1, alpha2, beta1, beta2, and gamma) as predictors on the measure of the paranormal belief as the criterion. In addition, we used model 4 of the PROCESS macro in SPSS^99^ to examine whether the effect of paranormal beliefs on inhibitory control (error in No-Go trials in Go/No-Go task) was mediated by frequency bands (delta, theta, alpha, alpha1, alpha2, beta, beta1, beta2, and gamma) in the whole brain (the first mediation model) and frontal lobe (the second mediation model) separately. The statistical significance of the indirect mediation effect on inhibitory control was assessed by bootstrapping (5000 samples) with a 95% confidence interval^99^. Statistical analyses were performed using IBM SPSS Statistics version 24 (IBM Inc., New York, USA), and MATLAB 2021a (MathWorks, Natick, Massachusetts), and p-values less than 0.05 were considered statistically significant.

## Results

### Behavioral performance in Go/No-Go task and demographic information

**Figure 2:**
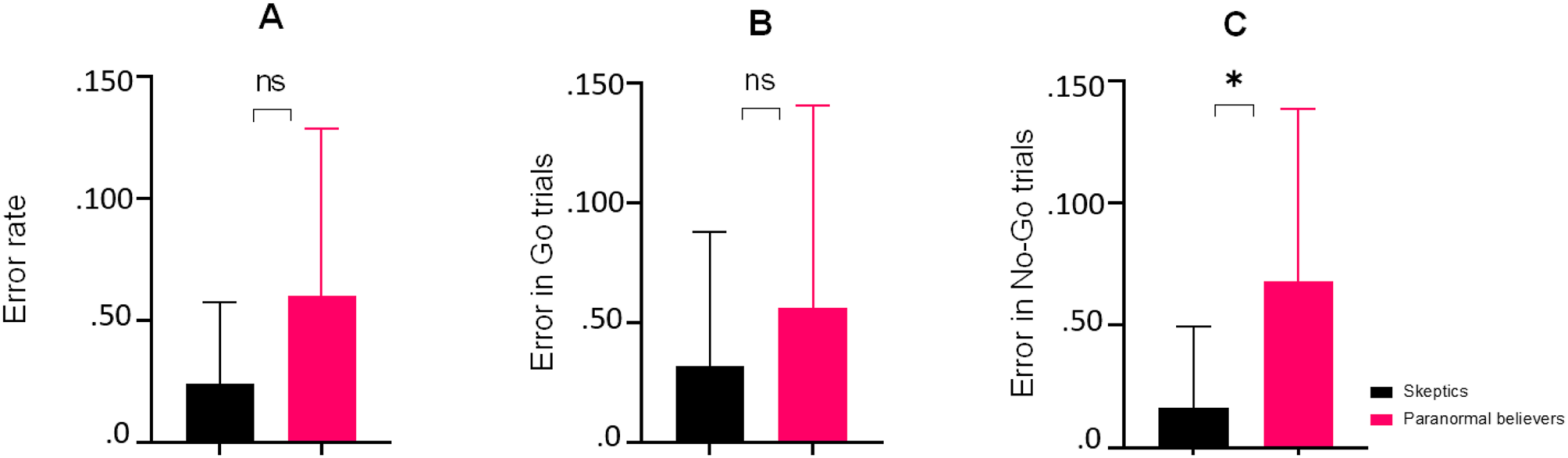
The behavioral performance in the paranormal believers and skeptics groups on the Go/No-Go task in (**A**) error rate, (**B**) error in Go trials, and error in No-Go trials. The error bars are SD. The black bar charts and red bar charts are for skeptics and paranormal believers groups respectively. *P < 0.05.

As can be seen in Table 1, No significant differences in age, sex, and education were observed between groups. However, paranormal believers had higher scores on the RPBS than skeptics. In addition, the CFQ result showed that paranormal believers reported significantly more general cognitive failures. The paranormal believers showed more errors in No-Go trials (p = 0.04) and errors in Go trials (p = 0.46) in the Go/No-Go task compared with the skeptics (Fig. 2).

**Table 1:** Demographic and behavioral characteristics of the participants.

Variables	Paranormal believers (N = 10)	Skeptics (N = 10)	t, χ^2^	P
Demographic data				
Age (years)	22.30 (3.94)	22.70 (4.39)	0.21^a^	0.83
M/F (number)	5/5	5/5	0.00^b^	1.00
Education (years)	14.90 (1.79)	15.40 (1.71)	− 0.63^a^	0.53
Behavioral data				
PRBS	84.80 (17.03)	35.30 (6.23)	− 8.62^a^	0.01
CFQ	36.70 (8.02)	25.30 (7.57)	− 3.26^a^	0.04
Go/No-Go task				
Error rate	0. 60 (0. 68)	0.24 (0.33)	− 1.48^a^	0.15
Error in Go	0. 56 (0. 84)	0.32 (0. 55)	− 0.74^a^	0.46
Error in No-Go	0. 68 (0. 70)	0.16 (0. 33)	− 2.10^a^	0.04

Data are given as mean (SD).

RPBS The Revised Paranormal Belief Scale; CFQ The Cognitive Failures Questionnaire.

^a^Independent sample *t*-test was used.

^b^Chi-square test was used.

### EEG oscillatory activity

**Figure 3:**
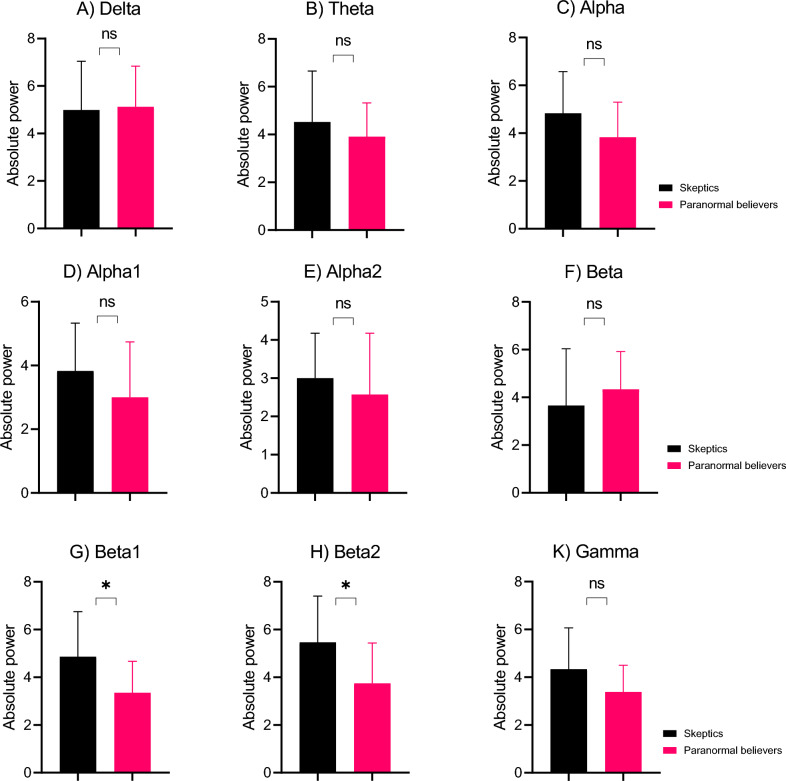
The absolute power of EEG frequency bands in the paranormal believers and skeptics groups in the whole brain. The figures show the absolute power of the bands of (**A**) delta, (**B**) theta, (**C**) alpha, (**D**) alpha1, (**E**) alpha2, (**F**) beta, (**G**) beta1, (**H**) beta2, and (**K**) gamma. The error bars are SD. The black bar charts and red bar charts are for the skeptics and paranormal believers groups, respectively. *P < 0.05.

**Figure 4:**
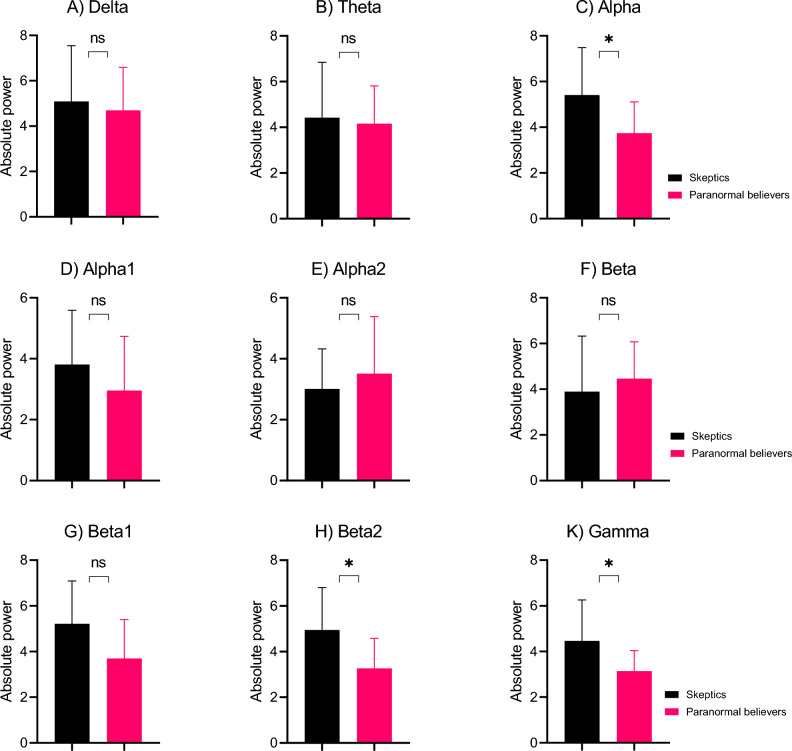
The absolute power of EEG frequency bands in the paranormal believers and skeptics groups in frontal lobe. The figures show the absolute power of the bands of (**A**) delta, (**B**) theta, (**C**) alpha, (**D**) alpha1, (**E**) alpha2, (**F**) beta, (**G**) beta1, (**H**) beta2, and (**K**) gamma. The error bars are SD. The black bar charts and red bar charts are for the skeptics and paranormal believers groups, respectively. *P < 0.05.

Figure 3 shows the two groups in terms of the absolute power in each band. In the whole brain, the independent t-test results showed significant differences between the groups for the absolute power in the beta1 (t_18_ = 2.117, p = 0.048, FDR corrected, Cohen's d = 0.997) and beta2 band activities (t_18_ = 2.075, p = 0.050, FDR corrected, Cohen's d = 0.978). As shown in Fig. 3, the absolute power in the beta1 and beta2 band activities of the paranormal believers was lower than that of the skeptics in the whole brain. In the frontal area, the independent t-test results showed a significant difference between the groups for the absolute power in the alpha (t_18_ = 2.123, p = 0.048, FDR corrected, Cohen's d = 1.00), beta2 (t_18_ = 2.341, p = 0.031, FDR corrected, Cohen's d = 1.103), and gamma-band activities (t_18_ = 2.090, p = 0.049, FDR corrected, Cohen's d = 0.985). As shown in Fig. 4, the absolute power in the alpha, beta2, and gamma-band activities of the paranormal believers was lower than that of the skeptics in the frontal area. In the right frontal area, the independent t-test results showed a significant difference between the groups for the absolute power in the beta1 (t_18_ = 2.548, p = 0.020, FDR corrected, Cohen's d = 1.201) and beta2 band activities (t_18_ = 2.680, p = 0.015, FDR corrected, Cohen's d = 1.263). The absolute power in the beta1 and beta2 band activities of the paranormal believers was lower than that of the skeptics in the right frontal area.

In the parietal area, the independent t-test results showed a significant difference between the groups for the absolute power in the beta1 (t_18_ = 2.216, p = 0.040, FDR corrected, Cohen's d = 1.044), and beta2 band activities (t_18_ = 2.133, p = 0.047, FDR corrected, Cohen's d = 1.005). The absolute power in the beta1 and beta2 band activities of the paranormal believers was lower than that of the skeptics in the parietal area. In the left parietal area, the independent t-test results showed a significant difference between the groups for the absolute power in the beta1 band activity (t_18_ = 2.335, p = 0.032, FDR corrected, Cohen's d = 1.100). The absolute power in the beta1 band activity of the paranormal believers was lower than that of the skeptics in the left parietal area. In the occipital area, the independent t-test results showed a significant difference between the groups for the absolute power in the beta1 (t_18_ = 2.494, p = 0.023, FDR corrected, Cohen's d = 1.175), and beta2 band activities (t_18_ = 2.086, p = 0.050, FDR corrected, Cohen's d = 0.983). The absolute power in the beta 1 and beta2 band activities of the paranormal believers was lower than that of the skeptics in the occipital area.

In the right hemisphere, the independent t-test results showed a significant difference between the groups for the absolute power in the beta1 band activity (t_18_ = 2.493, p = 0.023, FDR corrected, Cohen's d = 1.175). The absolute power in the beta1 band activity of the paranormal believers was lower than that of the skeptics in the right hemisphere. However, results showed no significant difference between the groups for the absolute power in other bands, areas, and hemispheres.

### Correlation between severity of paranormal beliefs and EEG oscillatory activity

**Figure 5:**
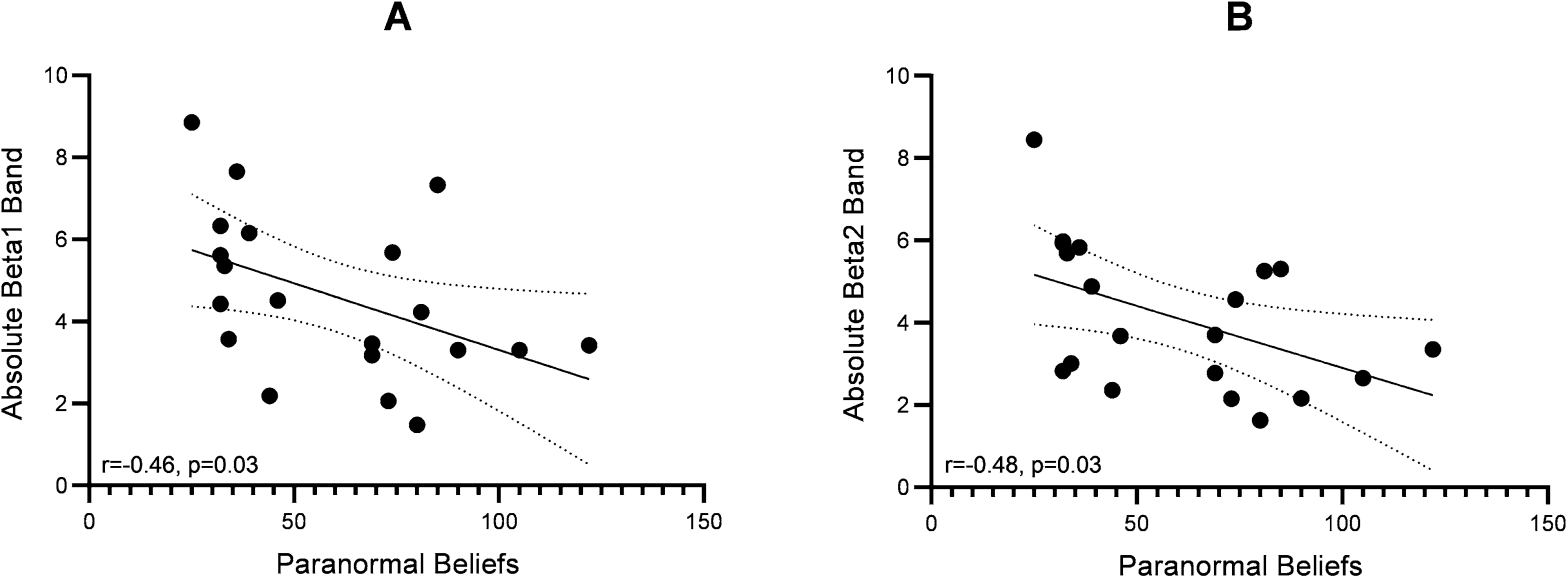
The figure of correlation between EEG power bands and paranormal beliefs score. The X-axis represents the scores of paranormal beliefs and the Y-axis the absolute power in (**A**) beta1, and (**B**) beta2 bands. Line dots are presented %95 confidence band of the best fit-line.

**Figure 6:**
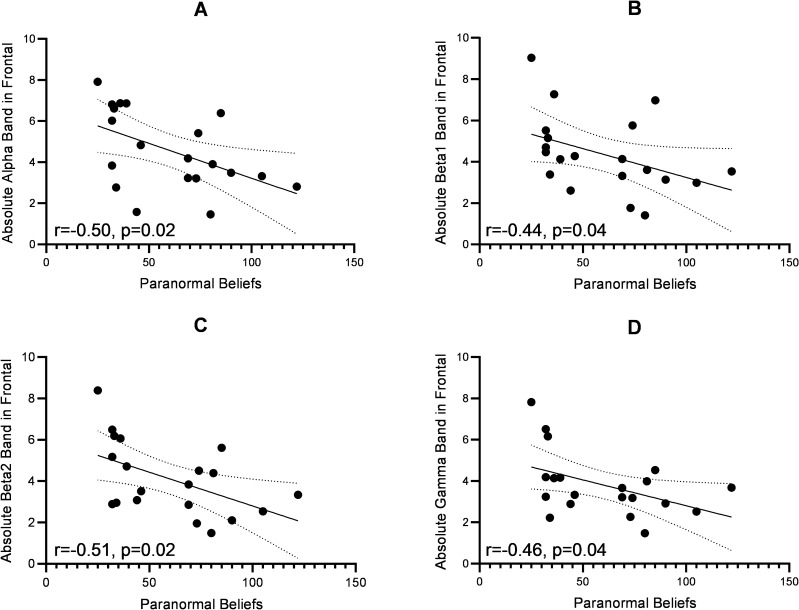
The figure of correlation between EEG power bands and paranormal beliefs score in frontal lobe. The X-axis represents the scores of paranormal beliefs and the Y-axis represents the absolute power in the (**A**) alpha, (**B**) beta1, (**C**) beta2, and (**D**) gamma band activities in frontal lobe. Line dots are presented %95 confidence band of the best fit-line.

In the whole brain, we found significant negative correlations between the severity of paranormal beliefs and absolute beta1(r = − 0.465, p = 0.039), and beta2 band activities (r = − 0.484, p = 0.031) (Fig. 5). In the frontal area, there were significant negative correlations between the severity of paranormal beliefs and absolute alpha (r = − 0.503, p = 0.024), beta1(r = − 0.449, p = 0.047), beta2(r = − 0.517, p = 0.020), gamma band activities (r = − 0.460, p = 0.041) (Fig. 6). In the right frontal area, the severity of paranormal beliefs was significantly negatively correlated with the alpha (r = − 0.471, p = 0.036), beta1 (r = − 0.547, p = 0.013), beta2 (r = − 0.568, p = 0.009), and gamma band activities (r = − 0.453, p = 0.045). In left frontal area, we found significant negative correlations between the severity of paranormal beliefs and absolute alpha band activities (r = − 0.455, p = 0.044).

In the parietal area, the severity of paranormal beliefs was significantly negatively correlated with absolute beta1(r = − 0.482, p = 0.032) and beta2 power activities (r = − 0.445, p = 0.049). In the left parietal area, there were significant negative correlations between the severity of paranormal beliefs and beta1 (r = − 0.496, p = 0.026) and beta2 band activities (r = − 0.449, p = 0.047).

In the left temporal area, there were significant negative correlations between the severity of paranormal beliefs and absolute beta2 band activity (r = − 0.471, p = 0.036). In addition, in the occipital area, there were significant negative correlations between the severity of paranormal beliefs and beta1 (r = − 0.507, p = 0.032) and beta2 band activities (r = − 0.471, p = 0.036). Additionally, in the right hemisphere, we found significant negative correlations between the severity of paranormal beliefs and absolute beta1 (r = − 0.516, p = 0.020), and beta2 band activities (r = − 0.444, p = 0.050). In the left hemisphere, we found significant negative correlations between the severity of paranormal beliefs and beta2 band activities (r = − 0.444, p = 0.050).

Additionally, the results of the multiple regression analysis show that the regression coefficient value (R) is 0.70 and the determination coefficient is (R2) 0.49. All frequency bands combined could explain 49% of the variance in paranormal beliefs. The amount of relative and effective contribution of the frequency bands (predictors), toward the dependent variable, namely the paranormal beliefs results, is presented in Table 2.

**Table 2:** Multiple regression analysis for paranormal beliefs based on frequency bands.

Predictor	B	SE	β	p
(Constant)	66.66	46.27		0.175
Delta	− 3.03	3.76	− 0.19	0.436
Theta	4.11	4.92	0.26	0.420
Alpha1	− 17.87	8.28	− 1.03	0.052
Alpha2	22.03	13.50	0.93	0.129
Beta1	3.08	9.69	0.21	0.756
Beta2	− 18.90	17.00	− 1.17	0.288
Gamma	12.66	12.62	0.67	0.335

*B* The unstandardized beta, *SE* standard error for the unstandardized beta, *β* standardized beta.

### Correlation between inhibitory control and EEG oscillatory activity

**Figure 7:**
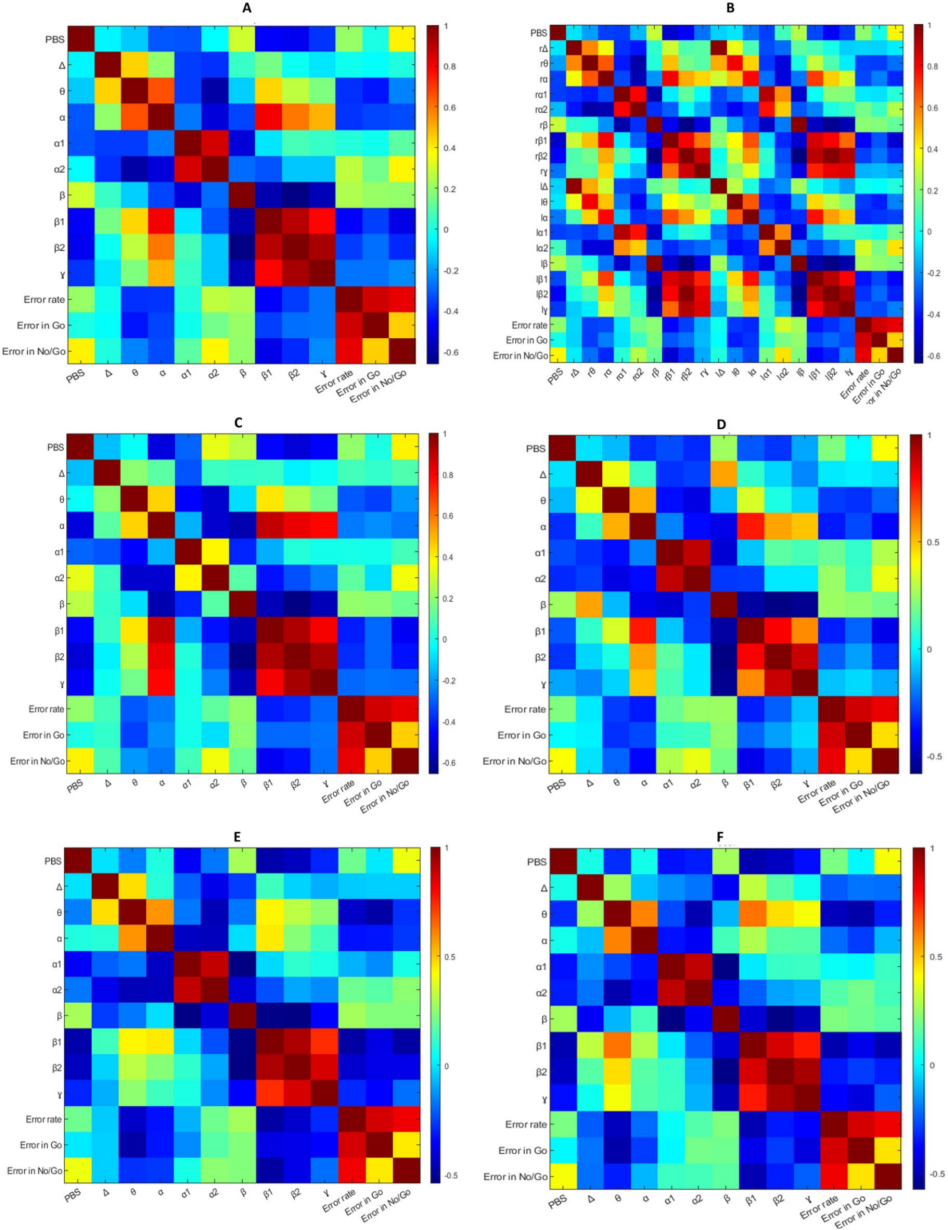
The heatmap results of pairwise correlation matrices of the frequency bands, RPBS, and Go-No/Go task in (**A**) whole brain, (**B**) right and left hemispheres (**C**) frontal lobe (**D**) temporal lobe (**E**) parietal lobe, and (**F**) occipital lobe. *RPBS* paranormal beliefs scale, *Δ* Delta band, *θ* Theta band, *α* Alpha band, *α1* Alpha1 band, *α2* Alpha2 band, *β* Beta band, *β1* Beta1 band, *β2* Beta2 band, *ɣ* Gamma band, *r* right hemisphere, *l* left hemisphere.

We also observed relationships between EEG frequency bands and the Go/No-Go task performance (Fig. 7, Supplementary Table 13). Error in No-Go trials in the Go/No-Go task was significantly negatively correlated with absolute power in the absolute beta1 power in the whole brain (r = − 0.494, p = 0.027), frontal area (r = − 0.473, p = 0.035), parietal area (r = − 0.490, p = 0.028), occipital area (r = − 0.498, p = 0.025), and right hemisphere (r = − 0. 512, p = 0.021). In addition, there was no significant correlation between Error in Go trials and absolute power in frequency bands.

### Model

**Figure 8:**
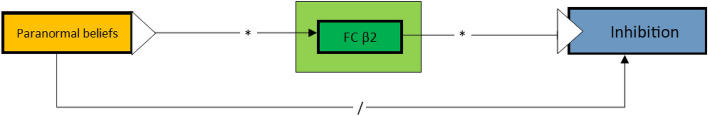
The mediation model. The mediation model tests the relationship between paranormal beliefs as an independent variable, inhibition (error in No-Go trials in Go/No-Go task) as a dependent variable, frequency beta2 band in frontal lobe as mediator. *FC* frontal cortex, *β* beta band, / insignificant, *Significant in p < 0.005.

Besides, a mediation model with inhibitory control (error in No-Go trials in Go/No-Go task) as a dependent variable, paranormal beliefs as an independent variable, frequency bands (delta, theta, alpha, alpha1, alpha2, beta, beta1, beta2, and gamma) as mediators was constructed for each band separately. The Hayes' PROCESS macro for SPSS was also used to test the mediation model^99^ (model 4, 5000 bootstrap resamples, 95% CI). The results in supplementary Fig 1. showed that the effect of paranormal beliefs on inhibitory control was not mediated through frequency bands (delta, theta, alpha, alpha1, alpha2, beta, beta1, beta2, and gamma). Additionally, in the second mediation model in frontal lobe, the present study assessed the mediating role of beta2 band oscillation on the relationship between paranormal beliefs and inhibitory control. The results revealed a significant indirect effect of impact of paranormal beliefs on Inhibitory control. Furthermore, the direct effect of paranormal beliefs on Inhibitory control in the presence of the beta2 band oscillation in frontal lobe (mediator) was not significant. Mediation schematic summary is shown in Fig. 8. In addition, the present study assessed the mediating role of other frequency bands (delta, theta, alpha, alpha1, alpha2, beta, beta1, and gamma) in the frontal lobe on the relationship between paranormal beliefs and inhibitory control. In another frequency bands, the results didn’t show a significant direct and indirect effect of impact of paranormal beliefs on Inhibitory control.

## Discussion

Our main results indicate that paranormal believers show reduced beta power compared to skeptics. Additionally, paranormal believers were more impaired in inhibitory control on the No-Go trials, as compared to the skeptics. As expected, lower absolute beta1 and beta2 power activities during resting-state EEG were shown in paranormal believers. Previous studies have shown that a weaker beta band activity in No-Go trials is associated with poor inhibitory control^42,45–47,58^. Beta band oscillations within the cognitive function occipito-frontal network is modulated by inhibitory control^100^. The top-down control of behavior has been used to illustrate the connection between cognitive processes and frontal beta oscillations^101^. Compared to instructed decisions, a study found that free decisions were accompanied by higher frontoparietal beta-band coherence^102^. Similar to the search and repetition phases of this task, free and instructed decisions demand differing levels of inhibitory control^44^. The "status quo" maintained by this proposed explanation is consistent with an extended theory of beta oscillations^43^. These authors suggest that when a task has a "strong endogenous top-down component," such activity should be connected to the active maintenance of a cognitive set in the cognitive domain. Furthermore, it's important to note that, like the majority of neurophysiological research on inhibitory control, our evidence for the relationship between beta power and inhibitory control is correlational. The causal mechanisms underlying these associations can only be shown by direct experimental changes in brain activity that concentrate on beta oscillations.

From the view of neurochemistry, dopamine (DA), which is essential for stabilizing brain networks^103^, is most likely a key player in schizophrenia psychopathology^104^. Furthermore, the DA levels in neural networks could affect the temporal dynamics of beta oscillations^105^. For instance, the subthalamic nucleus's beta-band increased when Parkinson’s patients were given the DA precursor Levodopa^106^. However, it is still speculative at this date to attribute the beta-band impairments in schizophrenia patients to the dopaminergic system. Future studies using combined EEG and positron emission tomography may look into the relationship between beta-band differences in schizophrenia patients and DA. Additionally, concerning the reduction of the beta power in paranormal believers compared to skeptics in the present study, we can refer to the relationship between paranormal experiences and the DA^107^, as well as lower activity in the rIFG area^63^. Moreover, beta band differences during EEG resting-state in the lack of stimulant may indicate dopamine depletion in the rIFG area^108^, which is related to inhibitory control. Additionally, a study found that less constrained firing rates were caused by enhanced dopaminergic transmission in mesolimbic neurons^109^, and would generate more internal noise and promote, on the phenomenal level, loosened associations, and paranormal beliefs^110^.

Our results showed that in terms of absolute gamma power, paranormal believers showed lower activation in the frontal lobe compared to skeptics. According to the theory of cognitive deficits in paranormal beliefs^3^, it is possible to assume that this cognitive deficit may be associated with differences in brain activity. Several studies showed a relationship between paranormal belief and schizotypy, and other characteristics associated with schizophrenia^1,111,112^. Therefore, studies focusing on paranormal phenomena have reported a reduction in gamma band in individuals with high transliminality^68^, hallucinations, positive schizophrenia symptoms^113^, and dysfunction in attention and cognition^114^. Gamma band activity has been related to a broad range of cognitive processes^52–55,115–118^. Therefore, widespread gamma power in cognitive functions, such as inhibitory control^39^, may be linked to the feature binding theory in the brain^119,120^. Given that the gamma band activity has decreased in paranormal believers in the frontal lobe compared to skeptics, this can also be attributed to the poor inhibitory control in these individuals. From the cognitive neuroscience perspective, the decrease in gamma band activity may be related to neural dysfunction in the frontal lobe^121^, reflecting impairment in the synchronous inhibition of pyramidal neurons^122^, and the GABAergic neurotransmission^123^. Furthermore, previous studies showed that the frontal lobe is involved in higher-order cognitive functions^124–126^. Concerning the frontal lobe dysfunction in paranormal beliefs^74^, the results of the present study also show that in paranormal believers as compared to skeptics, the absolute power of the beta was significantly reduced in the frontal area. These findings indicate that low gamma activity in the frontal lobe and low beta band activity in the frontal lobe may also be associated with the inhibitory control deficit in paranormal believers.

We also found that paranormal believers showed a low absolute alpha power in the bilateral frontal lobe compared to skeptics. Alpha power reflected a neural inhibitory mechanism involved in the influence of external sensory information at the resting-state EEG^127^. Through alpha power, this mechanism may be related to the GABAergic inhibitory activity via an inter-neuronal network in the brain^128^, and an unbalance between the GABAergic system and glutamatergic neurotransmission. Alpha power has been associated with the integrity of the cognitive process^129^, while it has been found to play a critical role in inhibitory control^130^. The alpha band differences may be associated with a range of information processing deficits such as inhibitory control^26,131^.

Our results indicate that paranormal believers showed more errors in both the Go and No-Go trials compared to skeptics. Still, there was only a significant group difference in No/Go trials in the Go/No-Go task (Fig. 2). It seems that association of paranormal belief and Go/No-Go task performance is specific for inhibitory control, but that future studies could further investigate general associations of paranormal belief with task performance. Our results also showed that error in No-Go trials in the Go/No-Go task was significantly negatively correlated with beta power in the whole brain. Studies have shown reduced beta power in No-Go trials in different populations^42,45–49^. Perhaps, deficiency in inhibitory control is associated with beta band reduction. Additionally, our results demonstrated that all frequency bands combined could explain 49% of the variance in paranormal beliefs, although the model was not significant. This may be due to the small sample size in the present study. However, there was no previous study on the relationship between paranormal beliefs and EEG oscillatory activity, these findings seem to prompt further investigations into correlations with EEG oscillatory activity in large sample size. Additionally, in mediation model analysis, beta2 frequency band activation in the frontal lobe (but not in the whole brain) mediated the relationship between paranormal belief and inhibitory control. However, there was no significant direct effect of paranormal beliefs on inhibitory control. The lack of significant correlations between paranormal beliefs and inhibitory control through frequency bands could in part be due to the small sample size. It may be revealed with larger populations and further studies are required with a large sample size.

We had several limitations in this study. Firstly, convenient sampling and selection of the sample from university students constituted one of the limitations of the present study. Based on these results, future studies could address associations of higher order frequency band oscillations with phenomena related to paranormal belief, such as schizophrenia. Secondly, the sample size in the present study was small. Thirdly, cognitive failures were assessed with a self-reported scale. Future studies using objective methods to assess cognitive failures level, including cognitive and behavioral function tasks, are needed. Another limitation of this study is that no resting EEG with closed eyes was measured. In addition, concerning the participation of women in this study, some studies have reported that performance in cognitive tasks related to the prefrontal cortex might be affected by the levels of estrogen and progesterone hormones^132,133^. Therefore, future studies are recommended to control the status of female participants. Finally, according to previous studies based on the greater tendency of women to paranormal beliefs^134,135^, and due to the small sample size of men and women in each group, it was impossible to examine their performance separately. Therefore, we suggest that gender differences be explored in future studies.

In summary, the present study shows that paranormal belief is related to the reduced power of the alpha, beta, and gamma frequency bands, and reduced inhibitory control. This study will provide new insights into the role of differences between believers and skeptics in brain activity in paranormal believers.

## Supplementary Information

Supplementary Information.

## Data Availability

The datasets used during the current study are available from the corresponding author on reasonable request.

## References

[1] Tobacyk J, Milford G (1983). Belief in paranormal phenomena: Assessment instrument development and implications for personality functioning. J. Pers. Soc. Psychol..

[2] Wilson JA (2018). Reducing pseudoscientific and paranormal beliefs in university students through a course in science and critical thinking. Sci. Educ..

[3] Irwin HJ (2009). The Psychology of Paranormal Belief: A Researcher's Handbook.

[4] Wain O, Spinella M (2007). Executive functions in morality, religion, and paranormal beliefs. Int. J. Neurosci..

[5] Hergovich A, Arendasy M (2005). Critical thinking ability and belief in the paranormal. Personality Individ. Differ..

[6] Musch J, Ehrenberg K (2002). Probability misjudgment, cognitive ability, and belief in the paranormal. Br. J. Psychol..

[7] Richards A, Hellgren MG, French CC (2014). Inattentional blindness, absorption, working memory capacity, and paranormal belief. Psychol. Conscious. Theory Res. Pract..

[8] Dagnall N, Parker A, Munley G (2007). Paranormal belief and reasoning. Personality Individ. Differ..

[9] Sarbin, T. R. The role of imagination in narrative construction. *Narrative analysis: Studying the development of individuals in society*, 5–20 (2004).

[10] White CJ, Baimel A, Norenzayan A (2021). How cultural learning and cognitive biases shape religious beliefs. Curr. Opin. Psychol..

[11] Narmashiri A, Sohrabi A, Hatami J (2018). Perceptual processing in paranormal beliefs: A study of reaction time and bias. Soc. Cogn..

[12] Narmashir A (2017). Perceptual-cognitive biases in relation to paranormal beliefs: A comparative study in brain lateralization groups. Neuropsychology.

[13] Van Elk M (2015). Perceptual biases in relation to paranormal and conspiracy beliefs. PLoS ONE.

[14] Willard AK, Norenzayan A (2013). Cognitive biases explain religious belief, paranormal belief, and belief in life’s purpose. Cognition.

[15] Buss DM (2005). The Evolutionary Psychology Handbook.

[16] Prike T, Arnold MM, Williamson P (2017). Psychics, aliens, or experience? Using the anomalistic belief scale to examine the relationship between type of belief and probabilistic reasoning. Conscious. Cogn..

[17] Svedholm AM, Lindeman M (2013). The separate roles of the reflective mind and involuntary inhibitory control in gatekeeping paranormal beliefs and the underlying intuitive confusions. Br. J. Psychol..

[18] Riekki, T. Neuro-cognitive factors contributing to paranormal beliefs: Core knowledge violations, cognitive inhibition, and the social brain. *University of Helsinki Institute of Behavioural Sciences Studies in Psychology* (2014).

[19] De Neys W (2012). Bias and conflict: A case for logical intuitions. Perspect. Psychol. Sci..

[20] Pennycook G, Cheyne JA, Seli P, Koehler DJ, Fugelsang JA (2012). Analytic cognitive style predicts religious and paranormal belief. Cognition.

[21] Gervais WM, Norenzayan A (2012). Analytic thinking promotes religious disbelief. Science.

[22] Miyake A (2000). The unity and diversity of executive functions and their contributions to complex “frontal lobe” tasks: A latent variable analysis. Cogn. Psychol..

[23] Hood B (2009). Supersense: From Superstition to Religion-The Brain Science of Belief.

[24] Hood BM, Lindeman M, Riekki T (2011). Is weaker inhibition associated with supernatural beliefs?. J. Cogn. Cult..

[25] Narmashiri A, Hatami J, Khosrowabadi R, Sohrabi A (2021). The role of cognitive control in paranormal beliefs: A study based on performance in go/no-go task. Basic Clin. Neurosci..

[26] Rogala J, Kublik E, Krauz R, Wróbel A (2020). Resting-state EEG activity predicts frontoparietal network reconfiguration and improved attentional performance. Sci. Rep..

[27] Buzsáki G, Anastassiou CA, Koch C (2012). The origin of extracellular fields and currents—EEG, ECoG, LFP and spikes. Nat. Rev. Neurosci..

[28] Anderson AJ, Perone S (2018). Developmental change in the resting state electroencephalogram: Insights into cognition and the brain. Brain Cogn..

[29] van Dongen-Boomsma M (2010). Relation between resting EEG to cognitive performance and clinical symptoms in adults with attention-deficit/hyperactivity disorder. Neurosci. Lett..

[30] Finnigan S, Robertson IH (2011). Resting EEG theta power correlates with cognitive performance in healthy older adults. Psychophysiology.

[31] Kounios J (2008). The origins of insight in resting-state brain activity. Neuropsychologia.

[32] Nguyen LT (2017). Theta and alpha alterations in amnestic mild cognitive impairment in semantic Go/NoGo tasks. Front. Aging Neurosci..

[33] Taylor MJ, Baldeweg T (2002). Application of EEG, ERP and intracranial recordings to the investigation of cognitive functions in children. Dev. Sci..

[34] Zafar, R. *et al.**014 IEEE Conference on Biomedical Engineering and Sciences (IECBES)*, 907–910 (IEEE).

[35] Kamarajan C (2004). The role of brain oscillations as functional correlates of cognitive systems: A study of frontal inhibitory control in alcoholism. Int. J. Psychophysiol..

[36] Rossini PM, Rossi S, Babiloni C, Polich J (2007). Clinical neurophysiology of aging brain: From normal aging to neurodegeneration. Prog. Neurobiol..

[37] Yamanaka K, Yamamoto Y (2010). Single-trial EEG power and phase dynamics associated with voluntary response inhibition. J. Cogn. Neurosci..

[38] Lee JY (2017). Resting-state EEG activity related to impulsivity in gambling disorder. J. Behav. Addict..

[39] Bresnahan SM, Barry RJ (2002). Specificity of quantitative EEG analysis in adults with attention deficit hyperactivity disorder. Psychiatry Res..

[40] Choi J-S (2013). Resting-state beta and gamma activity in Internet addiction. Int. J. Psychophysiol..

[41] Kühn AA (2004). Event-related beta desynchronization in human subthalamic nucleus correlates with motor performance. Brain.

[42] Zhang Y, Chen Y, Bressler SL, Ding M (2008). Response preparation and inhibition: The role of the cortical sensorimotor beta rhythm. Neuroscience.

[43] Engel AK, Fries P (2010). Beta-band oscillations: Signalling the status quo?. Curr. Opin. Neurobiol..

[44] Stoll FM (2016). The effects of cognitive control and time on frontal beta oscillations. Cereb. Cortex.

[45] Moliadze V (2020). Significance of beta-band oscillations in autism spectrum disorders during motor response inhibition tasks: A MEG study. Brain Topogr..

[46] Wheaton L, Fridman E, Bohlhalter S, Vorbach S, Hallett M (2009). Left parietal activation related to planning, executing and suppressing praxis hand movements. Clin. Neurophysiol..

[47] Wu H-M (2019). Attenuated NoGo-related beta desynchronisation and synchronisation in Parkinson’s disease revealed by magnetoencephalographic recording. Sci. Rep..

[48] Pani P (2014). Alpha-and beta-band oscillations subserve different processes in reactive control of limb movements. Front. Behav. Neurosci..

[49] Swann N (2009). Intracranial EEG reveals a time-and frequency-specific role for the right inferior frontal gyrus and primary motor cortex in stopping initiated responses. J. Neurosci..

[50] Tzagarakis C, Ince NF, Leuthold AC, Pellizzer G (2010). Beta-band activity during motor planning reflects response uncertainty. J. Neurosci..

[51] Yamano E, Ishii A, Tanaka M, Nomura S, Watanabe Y (2016). Neural basis of individual differences in the response to mental stress: A magnetoencephalography study. Brain Imaging Behav..

[52] Engel AK, Fries P, Singer W (2001). Dynamic predictions: oscillations and synchrony in top–down processing. Nat. Rev. Neurosci..

[53] Engel AK, König P, Kreiter AK, Schillen TB, Singer W (1992). Temporal coding in the visual cortex: new vistas on integration in the nervous system. Trends Neurosci..

[54] Fries P (2005). A mechanism for cognitive dynamics: Neuronal communication through neuronal coherence. Trends Cogn. Sci..

[55] Fries P (2009). Neuronal gamma-band synchronization as a fundamental process in cortical computation. Annu. Rev. Neurosci..

[56] Han Y-L (2020). Connectivity of the frontal cortical oscillatory dynamics underlying inhibitory control during a Go/No-Go task as a predictive biomarker in major depression. Front. Psych..

[57] Harmony T, Alba A, Marroquín JL, González-Frankenberger B (2009). Time-frequency-topographic analysis of induced power and synchrony of EEG signals during a Go/No-Go task. Int. J. Psychophysiol..

[58] Barry RJ (2010). Resting-state EEG gamma activity in children with attention-deficit/hyperactivity disorder. Clin. Neurophysiol..

[59] Benasich AA, Gou Z, Choudhury N, Harris KD (2008). Early cognitive and language skills are linked to resting frontal gamma power across the first 3 years. Behav. Brain Res..

[60] van Wingerden M, Vinck M, Lankelma JV, Pennartz CM (2010). Learning-associated gamma-band phase-locking of action–outcome selective neurons in orbitofrontal cortex. J. Neurosci..

[61] Chikazoe J, Konishi S, Asari T, Jimura K, Miyashita Y (2007). Activation of right inferior frontal gyrus during response inhibition across response modalities. J. Cogn. Neurosci..

[62] Hampshire A, Chamberlain SR, Monti MM, Duncan J, Owen AM (2010). The role of the right inferior frontal gyrus: Inhibition and attentional control. Neuroimage.

[63] Lindeman M, Svedholm AM, Riekki T, Raij T, Hari R (2013). Is it just a brick wall or a sign from the universe? An fMRI study of supernatural believers and skeptics. Soc. Cogn. Affect. Neurosci..

[64] Gianotti LR, Faber PL, Lehmann D (2022). International Congress Series.

[65] Pizzagalli D (2000). Brain electric correlates of strong belief in paranormal phenomena: Intracerebral EEG source and regional Omega complexity analyses. Psychiatry Res. Neuroimag..

[66] Brugger P, Mohr C (2008). The paranormal mind: How the study of anomalous experiences and beliefs may inform cognitive neuroscience. Cortex.

[67] Narmashiri A, Sohrabi A, Hatami J (2020). Brainwave pattern in paranormal beliefs: An EEG-based study in severe and mild groups. Neuropsychology.

[68] Fleck JI (2008). The transliminal brain at rest: Baseline EEG, unusual experiences, and access to unconscious mental activity. Cortex.

[69] Nash K, Kleinert T, Leota J, Scott A, Schimel J (2022). Resting-state networks of believers and non-believers: An EEG microstate study. Biol. Psychol..

[70] Kleinert, T. *et al.**A Self-controlled Mind Is Reflected by Stable Mental Processing*. (2022). 10.1177/09567976221110136 36279561

[71] Klimesch W, Sauseng P, Hanslmayr S (2007). EEG alpha oscillations: The inhibition–timing hypothesis. Brain Res. Rev..

[72] Alegre M, Alvarez-Gerriko I, Valencia M, Iriarte J, Artieda J (2008). Oscillatory changes related to the forced termination of a movement. Clin. Neurophysiol..

[73] Aron AR (2011). From reactive to proactive and selective control: Developing a richer model for stopping inappropriate responses. Biol. Psychiat..

[74] Cristofori I (2016). Neural correlates of mystical experience. Neuropsychologia.

[75] Narmashiri A, Hatami J, Khosrowabadi R, Sohrabi A (2022). EEG coherence during resting state over frontal regions in paranormal beliefs. Basic Clin. Neurosci..

[76] Tobacyk JJ (2004). A revised paranormal belief scale. Int. J. Transp. Stud..

[77] Vyse SA (2013). Believing in Magic: The Psychology of Superstition-Updated Edition.

[78] Riekki T, Lindeman M, Aleneff M, Halme A, Nuortimo A (2013). Paranormal and religious believers are more prone to illusory face perception than skeptics and non-believers. Appl. Cogn. Psychol..

[79] Drinkwater K, Denovan A, Dagnall N, Parker A (2017). An assessment of the dimensionality and factorial structure of the revised paranormal belief scale. Front. Psychol..

[80] Coleman EP, Croft RJ, Barkus E (2022). The profile of unusual beliefs associated with metacognitive thinking and attributional styles. PsyCh J..

[81] Broadbent DE, Cooper PF, FitzGerald P, Parkes KR (1982). The cognitive failures questionnaire (CFQ) and its correlates. Br. J. Clin. Psychol..

[82] Wallace JC, Vodanovich SJ (2003). Can accidents and industrial mishaps be predicted? Further investigation into the relationship between cognitive failure and reports of accidents. J. Bus. Psychol..

[83] Rast P, Zimprich D, Van Boxtel M, Jolles J (2009). Factor structure and measurement invariance of the cognitive failures questionnaire across the adult life span. Assessment.

[84] Bridger RS, Johnsen SÅK, Brasher K (2013). Psychometric properties of the cognitive failures questionnaire. Ergonomics.

[85] Fillmore MT, Rush CR, Hays L (2006). Acute effects of cocaine in two models of inhibitory control: implications of non-linear dose effects. Addiction.

[86] Barry RJ, Clarke AR, Johnstone SJ, Magee CA, Rushby JA (2007). EEG differences between eyes-closed and eyes-open resting conditions. Clin. Neurophysiol..

[87] Patriat R (2013). The effect of resting condition on resting-state fMRI reliability and consistency: A comparison between resting with eyes open, closed, and fixated. Neuroimage.

[88] Koshiyama D (2020). Neurophysiologic characterization of resting state connectivity abnormalities in schizophrenia patients. Front. Psych..

[89] Laptinskaya D (2020). Global EEG coherence as a marker for cognition in older adults at risk for dementia. Psychophysiology.

[90] Kaiser, D. & Sterman, M. *7th Annual Summer Sleep Workshop Multi-Site Training Program for Basic Sleep Research Lake Arrowhead, California.*

[91] Veltmeyer MD (2006). Integrative assessment of brain function in PTSD: brain stability and working memory. J. Integr. Neurosci..

[92] Hanslmayr S (2013). Enhanced resting-state oscillations in schizophrenia are associated with decreased synchronization during inattentional blindness. Hum. Brain Mapp..

[93] Narayanan B (2014). Resting state electroencephalogram oscillatory abnormalities in schizophrenia and psychotic bipolar patients and their relatives from the bipolar and schizophrenia network on intermediate phenotypes study. Biol. Psychiat..

[94] Wang C (2020). Altered relation of resting-state alpha rhythm with blood oxygen level dependent signal in healthy aging: Evidence by EEG-fMRI fusion analysis. Clin. Neurophysiol..

[95] López Zunini RA, Thivierge J-P, Kousaie S, Sheppard C, Taler V (2013). Alterations in resting-state activity relate to performance in a verbal recognition task. PLoS ONE.

[96] Son K (2015). Neurophysiological features of Internet gaming disorder and alcohol use disorder: A resting-state EEG study. Transl. Psychiatry.

[97] Lai CQ, Ibrahim H, Abdullah MZ, Azman A, Abdullah JM (2020). Detection of moderate traumatic brain injury from resting-state eye-closed electroencephalography. Comput. Intell. Neurosci..

[98] Benjamini Y, Yekutieli D (2001). The control of the false discovery rate in multiple testing under dependency. Ann. Stat..

[99] Hayes, A. F. (University of Kansas, 2012).

[100] Tzagarakis C, Thompson A, Rogers RD, Pellizzer G (2019). The degree of modulation of beta band activity during motor planning is related to trait impulsivity. Front. Integr. Neurosci..

[101] Buschman TJ, Miller EK (2007). Top-down versus bottom-up control of attention in the prefrontal and posterior parietal cortices. Science.

[102] Pesaran B, Nelson MJ, Andersen RA (2008). Free choice activates a decision circuit between frontal and parietal cortex. Nature.

[103] Kyaga S (2013). Mental illness, suicide and creativity: 40-year prospective total population study. J. Psychiatr. Res..

[104] Heinz A (2002). Dopaminergic dysfunction in alcoholism and schizophrenia–psychopathological and behavioral correlates. Eur. Psychiatry.

[105] Moran JK, Michail G, Heinz A, Keil J, Senkowski D (2019). Long-range temporal correlations in resting state beta oscillations are reduced in schizophrenia. Front. Psych..

[106] Prell T, Lautenschläger J, Witte O, Carri M, Grosskreutz J (2012). The unfolded protein response in models of human mutant G93A amyotrophic lateral sclerosis. Eur. J. Neurosci..

[107] Brugger P, Graves R (1997). Right hemispatial inattention and magical ideation. Eur. Arch. Psychiatry Clin. Neurosci..

[108] George JS (2013). Dopaminergic therapy in Parkinson's disease decreases cortical beta band coherence in the resting state and increases cortical beta band power during executive control. NeuroImage Clin..

[109] Krummenacher P, Mohr C, Haker H, Brugger P (2010). Dopamine, paranormal belief, and the detection of meaningful stimuli. J. Cogn. Neurosci..

[110] Shaner A (1999). Delusions, superstitious conditioning and chaotic dopamine neurodynamics. Med. Hypotheses.

[111] Thalbourne MA (1994). Belief in the paranormal and its relationship to schizophrenia-relevant measures: A confirmatory study. Br. J. Clin. Psychol..

[112] Irwin HJ (1993). Belief in the paranormal: A review of the empirical literature. J. Am. Soc. Psychical Res..

[113] Herrmann C, Demiralp T (2005). Human EEG gamma oscillations in neuropsychiatric disorders. Clin. Neurophysiol..

[114] Fell J, Fernandez G, Klaver P, Elger CE, Fries P (2003). Is synchronized neuronal gamma activity relevant for selective attention?. Brain Res. Rev..

[115] Singer W, Gray CM (1995). Visual feature integration and the temporal correlation hypothesis. Annu. Rev. Neurosci..

[116] Senkowski D, Schneider TR, Foxe JJ, Engel AK (2008). Crossmodal binding through neural coherence: implications for multisensory processing. Trends Neurosci..

[117] Knyazev GG (2007). Motivation, emotion, and their inhibitory control mirrored in brain oscillations. Neurosci. Biobehav. Rev..

[118] Jensen O, Kaiser J, Lachaux J-P (2007). Human gamma-frequency oscillations associated with attention and memory. Trends Neurosci..

[119] Tallon-Baudry C (2003). Oscillatory synchrony and human visual cognition. J. Physiol..

[120] Tallon-Baudry C, Bertrand O, Hénaff M-A, Isnard J, Fischer C (2005). Attention modulates gamma-band oscillations differently in the human lateral occipital cortex and fusiform gyrus. Cereb. Cortex.

[121] Rosanova M (2009). Natural frequencies of human corticothalamic circuits. J. Neurosci..

[122] Gonzalez-Burgos G, Lewis DA (2008). GABA neurons and the mechanisms of network oscillations: implications for understanding cortical dysfunction in schizophrenia. Schizophr. Bull..

[123] Lewis DA, Hashimoto T, Volk DW (2005). Cortical inhibitory neurons and schizophrenia. Nat. Rev. Neurosci..

[124] Denes G, Signorini M, Volpato C (2005). Post graphemic impairments of writing: The case of micrographia. Neurocase.

[125] Fuster JM (2002). Frontal lobe and cognitive development. J. Neurocytol..

[126] Fang S, Wang Y, Jiang T (2016). The influence of frontal lobe tumors and surgical treatment on advanced cognitive functions. World Neurosurgery.

[127] Mathewson KE (2011). Pulsed out of awareness: EEG alpha oscillations represent a pulsed-inhibition of ongoing cortical processing. Front. Psychol..

[128] Jensen O, Mazaheri A (2010). Shaping functional architecture by oscillatory alpha activity: Gating by inhibition. Front. Hum. Neurosci..

[129] Klimesch W (1999). EEG alpha and theta oscillations reflect cognitive and memory performance: A review and analysis. Brain Res. Rev..

[130] Sauseng P, Griesmayr B, Freunberger R, Klimesch W (2010). Control mechanisms in working memory: A possible function of EEG theta oscillations. Neurosci. Biobehav. Rev..

[131] Van der Molen M (2012). Auditory change detection in fragile X syndrome males: A brain potential study. Clin. Neurophysiol..

[132] Amin Z, Epperson CN, Constable RT, Canli T (2006). Effects of estrogen variation on neural correlates of emotional response inhibition. Neuroimage.

[133] Solis-Ortiz S, Guevara M, Corsi-Cabrera M (2004). Performance in a test demanding prefrontal functions is favored by early luteal phase progesterone: An electroencephalographic study. Psychoneuroendocrinology.

[134] Narmashiri A, Sohrabi A, Hatami J, Amirfakhraei A, Haghighat S (2019). Investigating the role of brain lateralization and gender in paranormal beliefs. Basic Clin. Neurosci..

[135] Schulter G, Papousek I (2008). Believing in paranormal phenomena: Relations to asymmetry of body and brain. Cortex.

