# Author Correction: Paranormal believers show reduced resting EEG beta band oscillations and inhibitory control than skeptics

**DOI:** 10.1038/s41598-023-39133-2

**Published:** 2023-07-25

**Authors:** Abdolvahed Narmashiri, Javad Hatami, Reza Khosrowabadi, Ahmad Sohrabi

**Affiliations:** 1 School of Cognitive Sciences, Institute for Research in Fundamental Sciences (IPM), Tehran, Iran; 2 Bio-Intelligence Research Unit, Sharif Brain Center, Electrical Engineering Department, Sharif University of Technology, Tehran, Iran; 3 Shahid Beheshti University, Tehran, Iran; 4 University of Tehran, Tehran, Iran; 5 University of Kurdistan, Sanandaj, Iran

Correction to: *Scientific Reports* 10.1038/s41598-023-30457-7, published online 24 February 2023

The original version of this Article contained errors in Table 1. In column ‘t, χ^2^’ the superscript symbols, indicating whether the Independent sample t-test or the Chi-square test was used, were incorrect. The correct and incorrect values appear below.

Incorrect:

**Table:** 

Variables	Paranormal believers (N = 10)	Skeptics (N = 10)	t, χ^2^	P
Demographic data				
Age (years)	22.30 (3.94)	22.70 (4.39)	0.21	0.83
M/F (number)	5/5	5/5	0.00^a^	1.00
Education (years)	14.90 (1.79)	15.40 (1.71)	− 0.63	0.53
Behavioral data				
PRBS	84.80 (17.03)	35.30 (6.23)	− 8.62	0.01
CFQ	36.70 (8.02)	25.30 (7.57)	− 3.26	0.04
Go/No-Go task				
Error rate	0.60 (0.68)	0.24 (0.33)	− 1.48	0.15
Error in Go	0.56 (0.84)	0.32 (0.55)	− 0.74	0.46
Error in No-Go	0.68 (0.70)	0.16 (0.33)	− 2.10	0.04

Correct:

**Table:** 

Variables	Paranormal believers (N = 10)	Skeptics (N = 10)	t, χ^2^	P
Demographic data				
Age (years)	22.30 (3.94)	22.70 (4.39)	0.21^a^	0.83
M/F (number)	5/5	5/5	0.00^b^	1.00
Education (years)	14.90(1.79)	15.40 (1.71)	− 0.63^a^	0.53
Behavioral data				
PRBS	84.80 (17.03)	35.30 (6.23)	− 8.62^a^	0.01
CFQ	36.70 (8.02)	25.30 (7.57)	− 3.26^a^	0.04
Go/No-Go task				
Error rate	0.60 (0.68)	0.24 (0.33)	− 1.48^a^	0.15
Error in Go	0.56 (0.84)	0.32 (0.55)	− 0.74^a^	0.46
Error in No-Go	0.68 (0.70)	0.16 (0.33)	− 2.10^a^	0.04

As a result, the caption of Table 1 has been corrected to ‘Demographic and behavioral characteristics of the participants.’

The original Article has been corrected.

