# Supplementary material for: Paranormal believers show reduced resting EEG beta band oscillations and inhibitory control than skeptics
Source: Sci Rep. 2023 Feb 24;13:3258. doi: 10.1038/s41598-023-30457-7 (PMC9958009; doi:10.1038/s41598-023-30457-7)

| **Supplementary Table 1.** Pearson correlation between paranormal beliefs and EEG bands power in whole brain | | | | | | | | | | | | |
| --- | --- | --- | --- | --- | --- | --- | --- | --- | --- | --- | --- | --- |
|  | | 1 | 2 | 3 | 4 | 5 | 6 | 7 | 8 | 9 | 10 |  |
| 1.Paranormal beliefs | Pearson Correlation | 1 | -.057 | -.128 | -.312 | -.324 | .492^*^ | -.484^*^ | -.465^*^ | .295 | -.372 |  |
|  | Sig. (2-tailed) |  | .813 | .592 | .181 | .163 | .027 | .031 | .039 | .206 | .106 |  |
|  | N | 20 | 20 | 20 | 20 | 20 | 20 | 20 | 20 | 20 | 20 |  |
| 2.delta | Pearson Correlation | -.057 | 1 | .447^*^ | .199 | -.361 | -.015 | -.022 | .150 | .050 | -.078 |  |
|  | Sig. (2-tailed) | .813 |  | .048 | .401 | .118 | .949 | .928 | .527 | .836 | .744 |  |
|  | N | 20 | 20 | 20 | 20 | 20 | 20 | 20 | 20 | 20 | 20 |  |
| 3.theta | Pearson Correlation | -.128 | .447^*^ | 1 | .659^**^ | -.358 | -.145 | .269 | .436 | -.119 | .168 |  |
|  | Sig. (2-tailed) | .592 | .048 |  | .002 | .121 | .543 | .252 | .055 | .619 | .478 |  |
|  | N | 20 | 20 | 20 | 20 | 20 | 20 | 20 | 20 | 20 | 20 |  |
| 4.alpha | Pearson Correlation | -.312 | .199 | .659^**^ | 1 | -.264 | -.296 | .597^**^ | .807^**^ | -.349 | .497^*^ |  |
|  | Sig. (2-tailed) | .181 | .401 | .002 |  | .260 | .206 | .005 | .000 | .132 | .026 |  |
|  | N | 20 | 20 | 20 | 20 | 20 | 20 | 20 | 20 | 20 | 20 |  |
| 5.alpha1 | Pearson Correlation | -.324 | -.361 | -.358 | -.264 | 1 | -.468^*^ | .075 | -.081 | -.523^*^ | .047 |  |
|  | Sig. (2-tailed) | .163 | .118 | .121 | .260 |  | .037 | .754 | .735 | .018 | .843 |  |
|  | N | 20 | 20 | 20 | 20 | 20 | 20 | 20 | 20 | 20 | 20 |  |
| 6.alpha2 | Pearson Correlation | .492^*^ | -.015 | -.145 | -.296 | -.468^*^ | 1 | -.262 | -.256 | .431 | -.265 |  |
|  | Sig. (2-tailed) | .027 | .949 | .543 | .206 | .037 |  | .264 | .275 | .058 | .259 |  |
|  | N | 20 | 20 | 20 | 20 | 20 | 20 | 20 | 20 | 20 | 20 |  |
| 7.beta2 | Pearson Correlation | -.484^*^ | -.022 | .269 | .597^**^ | .075 | -.262 | 1 | .908^**^ | -.662^**^ | .931^**^ |  |
|  | Sig. (2-tailed) | .031 | .928 | .252 | .005 | .754 | .264 |  | .000 | .001 | .000 |  |
|  | N | 20 | 20 | 20 | 20 | 20 | 20 | 20 | 20 | 20 | 20 |  |
| 8.beta1 | Pearson Correlation | -.465^*^ | .150 | .436 | .807^**^ | -.081 | -.256 | .908^**^ | 1 | -.590^**^ | .782^**^ |  |
|  | Sig. (2-tailed) | .039 | .527 | .055 | .000 | .735 | .275 | .000 |  | .006 | .000 |  |
|  | N | 20 | 20 | 20 | 20 | 20 | 20 | 20 | 20 | 20 | 20 |  |
| 9.beta | Pearson Correlation | .295 | .050 | -.119 | -.349 | -.523^*^ | .431 | -.662^**^ | -.590^**^ | 1 | -.574^**^ |  |
|  | Sig. (2-tailed) | .206 | .836 | .619 | .132 | .018 | .058 | .001 | .006 |  | .008 |  |
|  | N | 20 | 20 | 20 | 20 | 20 | 20 | 20 | 20 | 20 | 20 |  |
| 10.gama | Pearson Correlation | -.372 | -.078 | .168 | .497^*^ | .047 | -.265 | .931^**^ | .782^**^ | -.574^**^ | 1 |  |
|  | Sig. (2-tailed) | .106 | .744 | .478 | .026 | .843 | .259 | .000 | .000 | .008 |  |  |
|  | N | 20 | 20 | 20 | 20 | 20 | 20 | 20 | 20 | 20 | 20 |  |
| *. Correlation is significant at the 0.05 level (2-tailed). | | | | | | | | | | | | |
| **. Correlation is significant at the 0.01 level (2-tailed). | | | | | | | | | | | | |

| **Supplementary Table 2.** Pearson correlation between paranormal beliefs and EEG bands power in frontal lobe | | | | | | | | | | | |
| --- | --- | --- | --- | --- | --- | --- | --- | --- | --- | --- | --- |
|  | | 1 | 2 | 3 | 4 | 5 | 6 | 7 | 8 | 9 | 10 |
| 1.Paranormal beliefs | Pearson Correlation | 1 | -.141 | -.025 | -.503^*^ | -.289 | .338 | -.517^*^ | -.449^*^ | .265 | -.460^*^ |
|  | Sig. (2-tailed) |  | .553 | .915 | .024 | .217 | .144 | .020 | .047 | .258 | .041 |
|  | N | 20 | 20 | 20 | 20 | 20 | 20 | 20 | 20 | 20 | 20 |
| 2.Frontal_Lobe_Delta_Mean | Pearson Correlation | -.141 | 1 | .187 | .108 | -.320 | .055 | -.060 | .050 | .048 | -.020 |
|  | Sig. (2-tailed) | .553 |  | .430 | .651 | .169 | .818 | .800 | .833 | .842 | .935 |
|  | N | 20 | 20 | 20 | 20 | 20 | 20 | 20 | 20 | 20 | 20 |
| 3.Frontal_Lobe_theta | Pearson Correlation | -.025 | .187 | 1 | .444^*^ | -.453^*^ | -.528^*^ | .255 | .415 | -.089 | .150 |
|  | Sig. (2-tailed) | .915 | .430 |  | .050 | .045 | .017 | .278 | .069 | .708 | .529 |
|  | N | 20 | 20 | 20 | 20 | 20 | 20 | 20 | 20 | 20 | 20 |
| 4.Frontal_Lobe_alpha | Pearson Correlation | -.503^*^ | .108 | .444^*^ | 1 | -.164 | -.531^*^ | .823^**^ | .890^**^ | -.586^**^ | .785^**^ |
|  | Sig. (2-tailed) | .024 | .651 | .050 |  | .489 | .016 | .000 | .000 | .007 | .000 |
|  | N | 20 | 20 | 20 | 20 | 20 | 20 | 20 | 20 | 20 | 20 |
| 5.Frontal_Lobe_alpha1 | Pearson Correlation | -.289 | -.320 | -.453^*^ | -.164 | 1 | .388 | .018 | -.172 | -.397 | -.008 |
|  | Sig. (2-tailed) | .217 | .169 | .045 | .489 |  | .091 | .940 | .470 | .083 | .974 |
|  | N | 20 | 20 | 20 | 20 | 20 | 20 | 20 | 20 | 20 | 20 |
| 6.Frontal_Lobe_alpha2 | Pearson Correlation | .338 | .055 | -.528^*^ | -.531^*^ | .388 | 1 | -.324 | -.431 | .100 | -.248 |
|  | Sig. (2-tailed) | .144 | .818 | .017 | .016 | .091 |  | .163 | .058 | .675 | .293 |
|  | N | 20 | 20 | 20 | 20 | 20 | 20 | 20 | 20 | 20 | 20 |
| 7.Frontal_Lobe_beta2 | Pearson Correlation | -.517^*^ | -.060 | .255 | .823^**^ | .018 | -.324 | 1 | .921^**^ | -.657^**^ | .931^**^ |
|  | Sig. (2-tailed) | .020 | .800 | .278 | .000 | .940 | .163 |  | .000 | .002 | .000 |
|  | N | 20 | 20 | 20 | 20 | 20 | 20 | 20 | 20 | 20 | 20 |
| 8.Frontal_Lobe_beta1 | Pearson Correlation | -.449^*^ | .050 | .415 | .890^**^ | -.172 | -.431 | .921^**^ | 1 | -.570^**^ | .805^**^ |
|  | Sig. (2-tailed) | .047 | .833 | .069 | .000 | .470 | .058 | .000 |  | .009 | .000 |
|  | N | 20 | 20 | 20 | 20 | 20 | 20 | 20 | 20 | 20 | 20 |
| 9.Frontal_Lobe_beta | Pearson Correlation | .265 | .048 | -.089 | -.586^**^ | -.397 | .100 | -.657^**^ | -.570^**^ | 1 | -.571^**^ |
|  | Sig. (2-tailed) | .258 | .842 | .708 | .007 | .083 | .675 | .002 | .009 |  | .008 |
|  | N | 20 | 20 | 20 | 20 | 20 | 20 | 20 | 20 | 20 | 20 |
| 10.Frontal_Lobe_gama | Pearson Correlation | -.460^*^ | -.020 | .150 | .785^**^ | -.008 | -.248 | .931^**^ | .805^**^ | -.571^**^ | 1 |
|  | Sig. (2-tailed) | .041 | .935 | .529 | .000 | .974 | .293 | .000 | .000 | .008 |  |
|  | N | 20 | 20 | 20 | 20 | 20 | 20 | 20 | 20 | 20 | 20 |
| *. Correlation is significant at the 0.05 level (2-tailed). | | | | | | | | | | | |
| **. Correlation is significant at the 0.01 level (2-tailed). | | | | | | | | | | | |

| **Supplementary Table 3.** Pearson correlation between paranormal beliefs and EEG bands power in temporal lobe | | | | | | | | | | | |
| --- | --- | --- | --- | --- | --- | --- | --- | --- | --- | --- | --- |
|  | | 1 | 2 | 3 | 4 | 5 | 6 | 7 | 8 | 9 | 10 |
| 1.Paranormal beliefs | Pearson Correlation | 1 | -.034 | -.100 | -.321 | -.274 | .523^*^ | -.310 | -.251 | .254 | -.127 |
|  | Sig. (2-tailed) |  | .887 | .676 | .168 | .242 | .018 | .184 | .286 | .281 | .592 |
|  | N | 20 | 20 | 20 | 20 | 20 | 20 | 20 | 20 | 20 | 20 |
| 2.Temporalal_Lobe_delta | Pearson Correlation | -.034 | 1 | .379 | .101 | -.317 | -.076 | -.053 | .089 | .543^*^ | -.179 |
|  | Sig. (2-tailed) | .887 |  | .099 | .672 | .174 | .749 | .824 | .708 | .013 | .449 |
|  | N | 20 | 20 | 20 | 20 | 20 | 20 | 20 | 20 | 20 | 20 |
| 3.Temporalal_Lobe_theta | Pearson Correlation | -.100 | .379 | 1 | .524^*^ | -.373 | -.162 | .049 | .341 | -.100 | -.063 |
|  | Sig. (2-tailed) | .676 | .099 |  | .018 | .105 | .495 | .836 | .142 | .673 | .792 |
|  | N | 20 | 20 | 20 | 20 | 20 | 20 | 20 | 20 | 20 | 20 |
| 4.Temporalal_Lobe_alpha | Pearson Correlation | -.321 | .101 | .524^*^ | 1 | -.184 | -.241 | .548^*^ | .765^**^ | -.429 | .495^*^ |
|  | Sig. (2-tailed) | .168 | .672 | .018 |  | .437 | .305 | .012 | .000 | .059 | .026 |
|  | N | 20 | 20 | 20 | 20 | 20 | 20 | 20 | 20 | 20 | 20 |
| 5.Temporalal_Lobe_alpha1 | Pearson Correlation | -.274 | -.317 | -.373 | -.184 | 1 | -.437 | .148 | -.083 | -.457^*^ | .119 |
|  | Sig. (2-tailed) | .242 | .174 | .105 | .437 |  | .054 | .532 | .729 | .043 | .618 |
|  | N | 20 | 20 | 20 | 20 | 20 | 20 | 20 | 20 | 20 | 20 |
| 6.Temporalal_Lobe_alpha2 | Pearson Correlation | .523^*^ | -.076 | -.162 | -.241 | -.437 | 1 | -.156 | -.131 | .368 | -.164 |
|  | Sig. (2-tailed) | .018 | .749 | .495 | .305 | .054 |  | .511 | .582 | .110 | .489 |
|  | N | 20 | 20 | 20 | 20 | 20 | 20 | 20 | 20 | 20 | 20 |
| 7.Temporalal_Lobe_beta2 | Pearson Correlation | -.310 | -.053 | .049 | .548^*^ | .148 | -.156 | 1 | .806^**^ | -.585^**^ | .885^**^ |
|  | Sig. (2-tailed) | .184 | .824 | .836 | .012 | .532 | .511 |  | .000 | .007 | .000 |
|  | N | 20 | 20 | 20 | 20 | 20 | 20 | 20 | 20 | 20 | 20 |
| 8.Temporalal_Lobe_beta1 | Pearson Correlation | -.251 | .089 | .341 | .765^**^ | -.083 | -.131 | .806^**^ | 1 | -.540^*^ | .579^**^ |
|  | Sig. (2-tailed) | .286 | .708 | .142 | .000 | .729 | .582 | .000 |  | .014 | .007 |
|  | N | 20 | 20 | 20 | 20 | 20 | 20 | 20 | 20 | 20 | 20 |
| 9.Temporalal_Lobe_beta | Pearson Correlation | .254 | .543^*^ | -.100 | -.429 | -.457^*^ | .368 | -.585^**^ | -.540^*^ | 1 | -.565^**^ |
|  | Sig. (2-tailed) | .281 | .013 | .673 | .059 | .043 | .110 | .007 | .014 |  | .009 |
|  | N | 20 | 20 | 20 | 20 | 20 | 20 | 20 | 20 | 20 | 20 |
| 10.Temporalal_Lobe_gama | Pearson Correlation | -.127 | -.179 | -.063 | .495^*^ | .119 | -.164 | .885^**^ | .579^**^ | -.565^**^ | 1 |
|  | Sig. (2-tailed) | .592 | .449 | .792 | .026 | .618 | .489 | .000 | .007 | .009 |  |
|  | N | 20 | 20 | 20 | 20 | 20 | 20 | 20 | 20 | 20 | 20 |
| *. Correlation is significant at the 0.05 level (2-tailed). | | | | | | | | | | | |
| **. Correlation is significant at the 0.01 level (2-tailed). | | | | | | | | | | | |

| **Supplementary Table 4.** Pearson correlation between paranormal beliefs and EEG bands power in parietal lobe | | | | | | | | | | | |
| --- | --- | --- | --- | --- | --- | --- | --- | --- | --- | --- | --- |
|  | | 1 | 2 | 3 | 4 | 5 | 6 | 7 | 8 | 9 | 10 |
| 1.Paranormal beliefs | Pearson Correlation | 1 | -.009 | -.161 | .079 | -.363 | -.172 | -.445^*^ | .285 | -.481^*^ | -.299 |
|  | Sig. (2-tailed) |  | .971 | .498 | .740 | .116 | .468 | .049 | .222 | .032 | .201 |
|  | N | 20 | 20 | 20 | 20 | 20 | 20 | 20 | 20 | 20 | 20 |
| 2.Parietal_Lobe_delta | Pearson Correlation | -.009 | 1 | .468^*^ | .095 | -.208 | -.383 | -.030 | -.264 | .127 | -.088 |
|  | Sig. (2-tailed) | .971 |  | .037 | .689 | .379 | .096 | .900 | .261 | .593 | .711 |
|  | N | 20 | 20 | 20 | 20 | 20 | 20 | 20 | 20 | 20 | 20 |
| 3.Parietal_Lobe_theta | Pearson Correlation | -.161 | .468^*^ | 1 | .583^**^ | -.172 | -.460^*^ | .317 | -.172 | .429 | .252 |
|  | Sig. (2-tailed) | .498 | .037 |  | .007 | .468 | .041 | .174 | .469 | .059 | .283 |
|  | N | 20 | 20 | 20 | 20 | 20 | 20 | 20 | 20 | 20 | 20 |
| 4.Parietal_Lobe_alpha | Pearson Correlation | .079 | .095 | .583^**^ | 1 | -.443 | -.455^*^ | .237 | -.015 | .447^*^ | .131 |
|  | Sig. (2-tailed) | .740 | .689 | .007 |  | .050 | .044 | .314 | .948 | .048 | .581 |
|  | N | 20 | 20 | 20 | 20 | 20 | 20 | 20 | 20 | 20 | 20 |
| 5.Parietal_Lobe_alpha1 | Pearson Correlation | -.363 | -.208 | -.172 | -.443 | 1 | .894^**^ | .105 | -.541^*^ | -.020 | .073 |
|  | Sig. (2-tailed) | .116 | .379 | .468 | .050 |  | .000 | .661 | .014 | .934 | .760 |
|  | N | 20 | 20 | 20 | 20 | 20 | 20 | 20 | 20 | 20 | 20 |
| 6.Parietal_Lobe_alpha2 | Pearson Correlation | -.172 | -.383 | -.460^*^ | -.455^*^ | .894^**^ | 1 | -.101 | -.413 | -.231 | -.140 |
|  | Sig. (2-tailed) | .468 | .096 | .041 | .044 | .000 |  | .671 | .071 | .327 | .557 |
|  | N | 20 | 20 | 20 | 20 | 20 | 20 | 20 | 20 | 20 | 20 |
| 7.Parietal_Lobe_beta2 | Pearson Correlation | -.445^*^ | -.030 | .317 | .237 | .105 | -.101 | 1 | -.537^*^ | .930^**^ | .865^**^ |
|  | Sig. (2-tailed) | .049 | .900 | .174 | .314 | .661 | .671 |  | .015 | .000 | .000 |
|  | N | 20 | 20 | 20 | 20 | 20 | 20 | 20 | 20 | 20 | 20 |
| 8.Parietal_Lobe_beta | Pearson Correlation | .285 | -.264 | -.172 | -.015 | -.541^*^ | -.413 | -.537^*^ | 1 | -.534^*^ | -.352 |
|  | Sig. (2-tailed) | .222 | .261 | .469 | .948 | .014 | .071 | .015 |  | .015 | .128 |
|  | N | 20 | 20 | 20 | 20 | 20 | 20 | 20 | 20 | 20 | 20 |
| 9.Parietal_Lobe_beta1 | Pearson Correlation | -.481^*^ | .127 | .429 | .447^*^ | -.020 | -.231 | .930^**^ | -.534^*^ | 1 | .738^**^ |
|  | Sig. (2-tailed) | .032 | .593 | .059 | .048 | .934 | .327 | .000 | .015 |  | .000 |
|  | N | 20 | 20 | 20 | 20 | 20 | 20 | 20 | 20 | 20 | 20 |
| 10.Parietal_Lobe_gama | Pearson Correlation | -.299 | -.088 | .252 | .131 | .073 | -.140 | .865^**^ | -.352 | .738^**^ | 1 |
|  | Sig. (2-tailed) | .201 | .711 | .283 | .581 | .760 | .557 | .000 | .128 | .000 |  |
|  | N | 20 | 20 | 20 | 20 | 20 | 20 | 20 | 20 | 20 | 20 |
| *. Correlation is significant at the 0.05 level (2-tailed). | | | | | | | | | | | |
| **. Correlation is significant at the 0.01 level (2-tailed). | | | | | | | | | | | |

| **Supplementary Table 5.** Pearson correlation between paranormal beliefs and EEG bands power in occipital lobe | | | | | | | | | | | |
| --- | --- | --- | --- | --- | --- | --- | --- | --- | --- | --- | --- |
|  | | 1 | 2 | 3 | 4 | 5 | 6 | 7 | 8 | 9 | 10 |
| 1.Paranormal beliefs | Pearson | 1 | .048 | -.311 | .042 | -.356 | -.337 | -.471^*^ | -.507^*^ | .262 | -.364 |
|  | Sig. (2- |  | .841 | .183 | .859 | .123 | .147 | .036 | .022 | .265 | .115 |
|  | N | 20 | 20 | 20 | 20 | 20 | 20 | 20 | 20 | 20 | 20 |
| 2.Occipital_Lobe_Delta | Pearson Correlation | .048 | 1 | .261 | -.085 | -.160 | -.203 | .150 | .310 | -.394 | .038 |
|  | Sig. (2-tailed) | .841 |  | .266 | .722 | .501 | .390 | .527 | .183 | .086 | .873 |
|  | N | 20 | 20 | 20 | 20 | 20 | 20 | 20 | 20 | 20 | 20 |
| 3.Occipital_Lobe_theta | Pearson Correlation | -.311 | .261 | 1 | .596^**^ | -.269 | -.505^*^ | .453^*^ | .623^**^ | -.100 | .392 |
|  | Sig. (2-tailed) | .183 | .266 |  | .006 | .251 | .023 | .045 | .003 | .675 | .087 |
|  | N | 20 | 20 | 20 | 20 | 20 | 20 | 20 | 20 | 20 | 20 |
| 4.Occipital_Lobe_alpha | Pearson Correlation | .042 | -.085 | .596^**^ | 1 | -.356 | -.395 | .148 | .282 | .116 | .139 |
|  | Sig. (2-tailed) | .859 | .722 | .006 |  | .123 | .084 | .533 | .229 | .628 | .559 |
|  | N | 20 | 20 | 20 | 20 | 20 | 20 | 20 | 20 | 20 | 20 |
| 5.Occipital_Lobe_alpha1 | Pearson Correlation | -.356 | -.160 | -.269 | -.356 | 1 | .898^**^ | .097 | -.018 | -.575^**^ | .106 |
|  | Sig. (2-tailed) | .123 | .501 | .251 | .123 |  | .000 | .683 | .941 | .008 | .655 |
|  | N | 20 | 20 | 20 | 20 | 20 | 20 | 20 | 20 | 20 | 20 |
| 6.Occipital_Lobe_alpha2 | Pearson Correlation | -.337 | -.203 | -.505^*^ | -.395 | .898^**^ | 1 | -.127 | -.235 | -.398 | -.106 |
|  | Sig. (2-tailed) | .147 | .390 | .023 | .084 | .000 |  | .594 | .319 | .082 | .657 |
|  | N | 20 | 20 | 20 | 20 | 20 | 20 | 20 | 20 | 20 | 20 |
| 7.Occipital_Lobe_beta2 | Pearson Correlation | -.471^*^ | .150 | .453^*^ | .148 | .097 | -.127 | 1 | .860^**^ | -.568^**^ | .938^**^ |
|  | Sig. (2-tailed) | .036 | .527 | .045 | .533 | .683 | .594 |  | .000 | .009 | .000 |
|  | N | 20 | 20 | 20 | 20 | 20 | 20 | 20 | 20 | 20 | 20 |
| 8.Occipital_Lobe_beta1 | Pearson Correlation | -.507^*^ | .310 | .623^**^ | .282 | -.018 | -.235 | .860^**^ | 1 | -.451^*^ | .763^**^ |
|  | Sig. (2-tailed) | .022 | .183 | .003 | .229 | .941 | .319 | .000 |  | .046 | .000 |
|  | N | 20 | 20 | 20 | 20 | 20 | 20 | 20 | 20 | 20 | 20 |
| 9.Occipital_Lobe_beta | Pearson Correlation | .262 | -.394 | -.100 | .116 | -.575^**^ | -.398 | -.568^**^ | -.451^*^ | 1 | -.494^*^ |
|  | Sig. (2-tailed) | .265 | .086 | .675 | .628 | .008 | .082 | .009 | .046 |  | .027 |
|  | N | 20 | 20 | 20 | 20 | 20 | 20 | 20 | 20 | 20 | 20 |
| 10.Occipital_Lobe_gama | Pearson Correlation | -.364 | .038 | .392 | .139 | .106 | -.106 | .938^**^ | .763^**^ | -.494^*^ | 1 |
|  | Sig. (2-tailed) | .115 | .873 | .087 | .559 | .655 | .657 | .000 | .000 | .027 |  |
|  | N | 20 | 20 | 20 | 20 | 20 | 20 | 20 | 20 | 20 | 20 |
| *. Correlation is significant at the 0.05 level (2-tailed). | | | | | | | | | | | |
| **. Correlation is significant at the 0.01 level (2-tailed). | | | | | | | | | | | |

**Supplementary Table 6.** Pearson correlation between paranormal beliefs and EEG bands power in right hemisphere

|  |  |  |  |  |  |  |  |  |  |  |  |  |  |  |  |  |  |  |  |  |  |  |  |  |  |  |  |  |  |  |  |  |  |  |  |  |  |  |  |
| --- | --- | --- | --- | --- | --- | --- | --- | --- | --- | --- | --- | --- | --- | --- | --- | --- | --- | --- | --- | --- | --- | --- | --- | --- | --- | --- | --- | --- | --- | --- | --- | --- | --- | --- | --- | --- | --- | --- | --- |
|  | | | 1 | 2 | | 3 | | | 4 | 5 | | 6 | | 7 | 8 | | 9 | | | 10 | | 11 | 12 | | 13 | 14 | | | 15 | | 16 | | 17 | | 18 | | 19 | | |
| 1.Paranormal beliefs | | 1 | | | -.065 | | -.182 | -.335 | | | -.341 | | -.140 | -.444^*^ | | -.516^*^ | | .289 | -.252 | | -.096 | | | -.065 | -.249 | | -.336 | .081 | | | | -.444^*^ | | -.322 | | .259 | | -.351 |  |
|  |  |  | | | .784 | | .443 | .149 | | | .142 | | .556 | .050 | | .020 | | .217 | .283 | | .688 | | | .784 | .289 | | .148 | .735 | | | | .050 | | .167 | | .271 | | .130 |  |
|  |  | 20 | | | 20 | | 20 | 20 | | | 20 | | 20 | 20 | | 20 | | 20 | 20 | | 20 | | | 20 | 20 | | 20 | 20 | | | | 20 | | 20 | | 20 | | 20 |  |
| 2.Right_delta | | -.065 | | | 1 | | .574^**^ | .361 | | | -.363 | | -.403 | .099 | | .299 | | .048 | .115 | | .972^**^ | | | .348 | .093 | | -.318 | -.268 | | | | .008 | | .093 | | .124 | | -.106 |  |
|  |  | .784 | | |  | | .008 | .118 | | | .116 | | .078 | .679 | | .201 | | .841 | .630 | | .000 | | | .133 | .696 | | .172 | .253 | | | | .972 | | .697 | | .601 | | .656 |  |
|  |  | 20 | | | 20 | | 20 | 20 | | | 20 | | 20 | 20 | | 20 | | 20 | 20 | | 20 | | | 20 | 20 | | 20 | 20 | | | | 20 | | 20 | | 20 | | 20 |  |
| 3.Right_theta | | -.182 | | | .574^**^ | | 1 | .652^**^ | | | -.403 | | -.581^**^ | .156 | | .484^*^ | | .030 | .113 | | .516^*^ | | | .810^**^ | .454^*^ | | -.347 | -.494^*^ | | | | .103 | | .285 | | .018 | | -.053 |  |
|  |  | .443 | | | .008 | |  | .002 | | | .078 | | .007 | .510 | | .030 | | .900 | .634 | | .020 | | | .000 | .045 | | .134 | .027 | | | | .666 | | .223 | | .941 | | .823 |  |
|  |  | 20 | | | 20 | | 20 | 20 | | | 20 | | 20 | 20 | | 20 | | 20 | 20 | | 20 | | | 20 | 20 | | 20 | 20 | | | | 20 | | 20 | | 20 | | 20 |  |
| 4.Right_alpha | | -.335 | | | .361 | | .652^**^ | 1 | | | -.323 | | -.558^*^ | .517^*^ | | .808^**^ | | -.202 | .446^*^ | | .295 | | | .481^*^ | .785^**^ | | -.259 | -.494^*^ | | | | .461^*^ | | .697^**^ | | -.249 | | .313 |  |
|  |  | .149 | | | .118 | | .002 |  | | | .165 | | .011 | .019 | | .000 | | .394 | .049 | | .206 | | | .032 | .000 | | .270 | .027 | | | | .041 | | .001 | | .290 | | .179 |  |
|  |  | 20 | | | 20 | | 20 | 20 | | | 20 | | 20 | 20 | | 20 | | 20 | 20 | | 20 | | | 20 | 20 | | 20 | 20 | | | | 20 | | 20 | | 20 | | 20 |  |
| 5.Right_alpha1 | | -.341 | | | -.363 | | -.403 | -.323 | | | 1 | | .799^**^ | .128 | | -.051 | | -.571^**^ | -.020 | | -.324 | | | -.247 | -.130 | | .915^**^ | .560^*^ | | | | .187 | | -.017 | | -.520^*^ | | .183 |  |
|  |  | .142 | | | .116 | | .078 | .165 | | |  | | .000 | .591 | | .831 | | .009 | .932 | | .163 | | | .294 | .584 | | .000 | .010 | | | | .429 | | .945 | | .019 | | .440 |  |
|  |  | 20 | | | 20 | | 20 | 20 | | | 20 | | 20 | 20 | | 20 | | 20 | 20 | | 20 | | | 20 | 20 | | 20 | 20 | | | | 20 | | 20 | | 20 | | 20 |  |
| 6.Right_alpha2 | | -.140 | | | -.403 | | -.581^**^ | -.558^*^ | | | .799^**^ | | 1 | -.017 | | -.284 | | -.363 | -.147 | | -.340 | | | -.449^*^ | -.362 | | .768^**^ | .468^*^ | | | | .018 | | -.226 | | -.300 | | .062 |  |
|  |  | .556 | | | .078 | | .007 | .011 | | | .000 | |  | .944 | | .225 | | .115 | .536 | | .142 | | | .047 | .116 | | .000 | .038 | | | | .939 | | .339 | | .198 | | .794 |  |
|  |  | 20 | | | 20 | | 20 | 20 | | | 20 | | 20 | 20 | | 20 | | 20 | 20 | | 20 | | | 20 | 20 | | 20 | 20 | | | | 20 | | 20 | | 20 | | 20 |  |
| 7.Right_beta2 | | -.444^*^ | | | .099 | | .156 | .517^*^ | | | .128 | | -.017 | 1 | | .803^**^ | | -.630^**^ | .873^**^ | | -.006 | | | .298 | .545^*^ | | .047 | -.308 | | | | .986^**^ | | .902^**^ | | -.640^**^ | | .916^**^ |  |
|  |  | .050 | | | .679 | | .510 | .019 | | | .591 | | .944 |  | | .000 | | .003 | .000 | | .980 | | | .201 | .013 | | .844 | .186 | | | | .000 | | .000 | | .002 | | .000 |  |
|  |  | 20 | | | 20 | | 20 | 20 | | | 20 | | 20 | 20 | | 20 | | 20 | 20 | | 20 | | | 20 | 20 | | 20 | 20 | | | | 20 | | 20 | | 20 | | 20 |  |
| 8.Right_beta1 | | -.516^*^ | | | .299 | | .484^*^ | .808^**^ | | | -.051 | | -.284 | .803^**^ | | 1 | | -.461^*^ | .608^**^ | | .197 | | | .380 | .665^**^ | | -.078 | -.378 | | | | .797^**^ | | .857^**^ | | -.513^*^ | | .625^**^ |  |
|  |  | .020 | | | .201 | | .030 | .000 | | | .831 | | .225 | .000 | |  | | .041 | .004 | | .406 | | | .099 | .001 | | .745 | .101 | | | | .000 | | .000 | | .021 | | .003 |  |
|  |  | 20 | | | 20 | | 20 | 20 | | | 20 | | 20 | 20 | | 20 | | 20 | 20 | | 20 | | | 20 | 20 | | 20 | 20 | | | | 20 | | 20 | | 20 | | 20 |  |
| 9.Right_beta | | .289 | | | .048 | | .030 | -.202 | | | -.571^**^ | | -.363 | -.630^**^ | | -.461^*^ | | 1 | -.486^*^ | | .138 | | | -.177 | -.337 | | -.527^*^ | -.240 | | | | -.610^**^ | | -.536^*^ | | .980^**^ | | -.558^*^ |  |
|  |  | .217 | | | .841 | | .900 | .394 | | | .009 | | .115 | .003 | | .041 | |  | .030 | | .560 | | | .455 | .146 | | .017 | .307 | | | | .004 | | .015 | | .000 | | .011 |  |
|  |  | 20 | | | 20 | | 20 | 20 | | | 20 | | 20 | 20 | | 20 | | 20 | 20 | | 20 | | | 20 | 20 | | 20 | 20 | | | | 20 | | 20 | | 20 | | 20 |  |
| 10.Right_gama | | -.252 | | | .115 | | .113 | .446^*^ | | | -.020 | | -.147 | .873^**^ | | .608^**^ | | -.486^*^ | 1 | | .028 | | | .179 | .337 | | .016 | -.272 | | | | .829^**^ | | .738^**^ | | -.482^*^ | | .845^**^ |  |
|  |  | .283 | | | .630 | | .634 | .049 | | | .932 | | .536 | .000 | | .004 | | .030 |  | | .906 | | | .449 | .146 | | .948 | .246 | | | | .000 | | .000 | | .031 | | .000 |  |
|  |  | 20 | | | 20 | | 20 | 20 | | | 20 | | 20 | 20 | | 20 | | 20 | 20 | | 20 | | | 20 | 20 | | 20 | 20 | | | | 20 | | 20 | | 20 | | 20 |  |
| 11.Left_delta | | -.096 | | | .972^**^ | | .516^*^ | .295 | | | -.324 | | -.340 | -.006 | | .197 | | .138 | .028 | | 1 | | | .273 | .026 | | -.277 | -.210 | | | | -.090 | | -.002 | | .235 | | -.183 |  |
|  |  | .688 | | | .000 | | .020 | .206 | | | .163 | | .142 | .980 | | .406 | | .560 | .906 | |  | | | .244 | .914 | | .237 | .374 | | | | .707 | | .993 | | .319 | | .441 |  |
|  |  | 20 | | | 20 | | 20 | 20 | | | 20 | | 20 | 20 | | 20 | | 20 | 20 | | 20 | | | 20 | 20 | | 20 | 20 | | | | 20 | | 20 | | 20 | | 20 |  |
| 12.Left_theta | | -.065 | | | .348 | | .810^**^ | .481^*^ | | | -.247 | | -.449^*^ | .298 | | .380 | | -.177 | .179 | | .273 | | | 1 | .648^**^ | | -.378 | -.421 | | | | .271 | | .438 | | -.174 | | .175 |  |
|  |  | .784 | | | .133 | | .000 | .032 | | | .294 | | .047 | .201 | | .099 | | .455 | .449 | | .244 | | |  | .002 | | .101 | .065 | | | | .248 | | .054 | | .464 | | .460 |  |
|  |  | 20 | | | 20 | | 20 | 20 | | | 20 | | 20 | 20 | | 20 | | 20 | 20 | | 20 | | | 20 | 20 | | 20 | 20 | | | | 20 | | 20 | | 20 | | 20 |  |
| 13.Left_alpha | | -.249 | | | .093 | | .454^*^ | .785^**^ | | | -.130 | | -.362 | .545^*^ | | .665^**^ | | -.337 | .337 | | .026 | | | .648^**^ | 1 | | -.276 | -.393 | | | | .533^*^ | | .766^**^ | | -.368 | | .459^*^ |  |
|  |  | .289 | | | .696 | | .045 | .000 | | | .584 | | .116 | .013 | | .001 | | .146 | .146 | | .914 | | | .002 |  | | .239 | .086 | | | | .016 | | .000 | | .110 | | .042 |  |
|  |  | 20 | | | 20 | | 20 | 20 | | | 20 | | 20 | 20 | | 20 | | 20 | 20 | | 20 | | | 20 | 20 | | 20 | 20 | | | | 20 | | 20 | | 20 | | 20 |  |
| 14.Left_alpha1 | | -.336 | | | -.318 | | -.347 | -.259 | | | .915^**^ | | .768^**^ | .047 | | -.078 | | -.527^*^ | .016 | | -.277 | | | -.378 | -.276 | | 1 | .573^**^ | | | | .068 | | -.147 | | -.487^*^ | | .080 |  |
|  |  | .148 | | | .172 | | .134 | .270 | | | .000 | | .000 | .844 | | .745 | | .017 | .948 | | .237 | | | .101 | .239 | |  | .008 | | | | .774 | | .535 | | .029 | | .736 |  |
|  |  | 20 | | | 20 | | 20 | 20 | | | 20 | | 20 | 20 | | 20 | | 20 | 20 | | 20 | | | 20 | 20 | | 20 | 20 | | | | 20 | | 20 | | 20 | | 20 |  |
| 15.Left_alpha2 | | .081 | | | -.268 | | -.494^*^ | -.494^*^ | | | .560^*^ | | .468^*^ | -.308 | | -.378 | | -.240 | -.272 | | -.210 | | | -.421 | -.393 | | .573^**^ | 1 | | | | -.251 | | -.342 | | -.226 | | -.150 |  |
|  |  | .735 | | | .253 | | .027 | .027 | | | .010 | | .038 | .186 | | .101 | | .307 | .246 | | .374 | | | .065 | .086 | | .008 |  | | | | .287 | | .140 | | .339 | | .527 |  |
|  |  | 20 | | | 20 | | 20 | 20 | | | 20 | | 20 | 20 | | 20 | | 20 | 20 | | 20 | | | 20 | 20 | | 20 | 20 | | | | 20 | | 20 | | 20 | | 20 |  |
| 16.Left_beta2 | | -.444^*^ | | | .008 | | .103 | .461^*^ | | | .187 | | .018 | .986^**^ | | .797^**^ | | -.610^**^ | .829^**^ | | -.090 | | | .271 | .533^*^ | | .068 | -.251 | | | | 1 | | .906^**^ | | -.633^**^ | | .931^**^ |  |
|  |  | .050 | | | .972 | | .666 | .041 | | | .429 | | .939 | .000 | | .000 | | .004 | .000 | | .707 | | | .248 | .016 | | .774 | .287 | | | |  | | .000 | | .003 | | .000 |  |
|  |  | 20 | | | 20 | | 20 | 20 | | | 20 | | 20 | 20 | | 20 | | 20 | 20 | | 20 | | | 20 | 20 | | 20 | 20 | | | | 20 | | 20 | | 20 | | 20 |  |
| 17.Left_beta1 | | -.322 | | | .093 | | .285 | .697^**^ | | | -.017 | | -.226 | .902^**^ | | .857^**^ | | -.536^*^ | .738^**^ | | -.002 | | | .438 | .766^**^ | | -.147 | -.342 | | | | .906^**^ | | 1 | | -.573^**^ | | .796^**^ |  |
|  |  | .167 | | | .697 | | .223 | .001 | | | .945 | | .339 | .000 | | .000 | | .015 | .000 | | .993 | | | .054 | .000 | | .535 | .140 | | | | .000 | |  | | .008 | | .000 |  |
|  |  | 20 | | | 20 | | 20 | 20 | | | 20 | | 20 | 20 | | 20 | | 20 | 20 | | 20 | | | 20 | 20 | | 20 | 20 | | | | 20 | | 20 | | 20 | | 20 |  |
| 18.Left_beta | | .259 | | | .124 | | .018 | -.249 | | | -.520^*^ | | -.300 | -.640^**^ | | -.513^*^ | | .980^**^ | -.482^*^ | | .235 | | | -.174 | -.368 | | -.487^*^ | -.226 | | | | -.633^**^ | | -.573^**^ | | 1 | | -.576^**^ |  |
|  |  | .271 | | | .601 | | .941 | .290 | | | .019 | | .198 | .002 | | .021 | | .000 | .031 | | .319 | | | .464 | .110 | | .029 | .339 | | | | .003 | | .008 | |  | | .008 |  |
|  |  | 20 | | | 20 | | 20 | 20 | | | 20 | | 20 | 20 | | 20 | | 20 | 20 | | 20 | | | 20 | 20 | | 20 | 20 | | | | 20 | | 20 | | 20 | | 20 |  |
| 19.Left_gama | | -.351 | | | -.106 | | -.053 | .313 | | | .183 | | .062 | .916^**^ | | .625^**^ | | -.558^*^ | .845^**^ | | -.183 | | | .175 | .459^*^ | | .080 | -.150 | | | | .931^**^ | | .796^**^ | | -.576^**^ | | 1 |  |
|  |  | .130 | | | .656 | | .823 | .179 | | | .440 | | .794 | .000 | | .003 | | .011 | .000 | | .441 | | | .460 | .042 | | .736 | .527 | | | | .000 | | .000 | | .008 | |  |  |
|  |  | 20 | | | 20 | | 20 | 20 | | | 20 | | 20 | 20 | | 20 | | 20 | 20 | | 20 | | | 20 | 20 | | 20 | 20 | | | | 20 | | 20 | | 20 | | 20 |  |
| *. Correlation is significant at the 0.05 level (2-tailed). | | | | | | | | | | | | | | | | | | | | | | | | | | | | | |  |  |  |  |  |  |  |  |  |  |
| **. Correlation is significant at the 0.01 level (2-tailed). | | | | | | | | | | | | | | | | | | | | | | | | | | | | | |  |  |  |  |  |  |  |  |  |  |

| **Supplementary Table 7.** Pearson correlation between paranormal beliefs and EEG bands power in left hemisphere | | | | | | | | | | | | | |
| --- | --- | --- | --- | --- | --- | --- | --- | --- | --- | --- | --- | --- | --- |
|  | | 1 | 2 | 3 | 4 | 5 | 6 | 7 | 8 | 9 | 10 | | 11 |
| 1.Paranormal beliefs | Pearson Correlation | 1 | -.161 | -.086 | .077 | -.471^*^ | -.339 | -.139 | .265 | -.547^*^ | -.568^**^ | | -.453^*^ |
|  | Sig. (2-tailed) |  | .499 | .720 | .747 | .036 | .144 | .558 | .260 | .013 | .009 | | .045 |
|  | N | 20 | 20 | 20 | 20 | 20 | 20 | 20 | 20 | 20 | 20 | | 20 |
| 2.Frontal_Lobe_Delta_Right | Pearson Correlation | -.161 | 1 | .306 | .256 | .202 | -.279 | -.304 | -.157 | .193 | .159 | | .225 |
|  | Sig. (2-tailed) | .499 |  | .189 | .277 | .394 | .233 | .193 | .509 | .415 | .504 | | .340 |
|  | N | 20 | 20 | 20 | 20 | 20 | 20 | 20 | 20 | 20 | 20 | | 20 |
| 3.Frontal_Lobe_theta_Right | Pearson Correlation | -.086 | .306 | 1 | .926^**^ | .429 | -.441 | -.557^*^ | -.152 | .419 | .260 | | .251 |
|  | Sig. (2-tailed) | .720 | .189 |  | .000 | .059 | .052 | .011 | .522 | .066 | .268 | | .287 |
|  | N | 20 | 20 | 20 | 20 | 20 | 20 | 20 | 20 | 20 | 20 | | 20 |
| 4.Frontal_Lobe_theta_Left | Pearson Correlation | .077 | .256 | .926^**^ | 1 | .308 | -.442 | -.485^*^ | -.141 | .353 | .199 | | .128 |
|  | Sig. (2-tailed) | .747 | .277 | .000 |  | .186 | .051 | .030 | .552 | .127 | .401 | | .589 |
|  | N | 20 | 20 | 20 | 20 | 20 | 20 | 20 | 20 | 20 | 20 | | 20 |
| 5.Frontal_Lobe_Alpha_Right | Pearson Correlation | -.471^*^ | .202 | .429 | .308 | 1 | -.264 | -.448^*^ | -.582^**^ | .875^**^ | .728^**^ | | .742^**^ |
|  | Sig. (2-tailed) | .036 | .394 | .059 | .186 |  | .261 | .047 | .007 | .000 | .000 | | .000 |
|  | N | 20 | 20 | 20 | 20 | 20 | 20 | 20 | 20 | 20 | 20 | | 20 |
| 6.Frontal_Lobe_Alpha1_Right | Pearson Correlation | -.339 | -.279 | -.441 | -.442 | -.264 | 1 | .919^**^ | -.235 | -.066 | .108 | | -.074 |
|  | Sig. (2-tailed) | .144 | .233 | .052 | .051 | .261 |  | .000 | .318 | .781 | .651 | | .757 |
|  | N | 20 | 20 | 20 | 20 | 20 | 20 | 20 | 20 | 20 | 20 | | 20 |
| 7.Frontal_Lobe_Alpha2_Right | Pearson Correlation | -.139 | -.304 | -.557^*^ | -.485^*^ | -.448^*^ | .919^**^ | 1 | -.105 | -.265 | -.031 | | -.244 |
|  | Sig. (2-tailed) | .558 | .193 | .011 | .030 | .047 | .000 |  | .659 | .260 | .898 | | .300 |
|  | N | 20 | 20 | 20 | 20 | 20 | 20 | 20 | 20 | 20 | 20 | | 20 |
| 8.Frontal_Lobe_beta_Right | Pearson Correlation | .265 | -.157 | -.152 | -.141 | -.582^**^ | -.235 | -.105 | 1 | -.577^**^ | -.662^**^ | | -.532^*^ |
|  | Sig. (2-tailed) | .260 | .509 | .522 | .552 | .007 | .318 | .659 |  | .008 | .001 | | .016 |
|  | N | 20 | 20 | 20 | 20 | 20 | 20 | 20 | 20 | 20 | 20 | | 20 |
| 9.Frontal_Lobe_beta1_Right | Pearson Correlation | -.547^*^ | .193 | .419 | .353 | .875^**^ | -.066 | -.265 | -.577^**^ | 1 | .877^**^ | | .745^**^ |
|  | Sig. (2-tailed) | .013 | .415 | .066 | .127 | .000 | .781 | .260 | .008 |  | .000 | | .000 |
|  | N | 20 | 20 | 20 | 20 | 20 | 20 | 20 | 20 | 20 | 20 | | 20 |
| 10.Frontal_Lobe_beta2_Right | Pearson Correlation | -.568^**^ | .159 | .260 | .199 | .728^**^ | .108 | -.031 | -.662^**^ | .877^**^ | 1 | | .871^**^ |
|  | Sig. (2-tailed) | .009 | .504 | .268 | .401 | .000 | .651 | .898 | .001 | .000 |  | | .000 |
|  | N | 20 | 20 | 20 | 20 | 20 | 20 | 20 | 20 | 20 | 20 | | 20 |
| 11.Frontal_Lobe_gama_Right | Pearson Correlation | -.453^*^ | .225 | .251 | .128 | .742^**^ | -.074 | -.244 | -.532^*^ | .745^**^ | .871^**^ | | 1 |
|  | Sig. (2-tailed) | .045 | .340 | .287 | .589 | .000 | .757 | .300 | .016 | .000 | .000 | |  |
|  | N | 20 | 20 | 20 | 20 | 20 | 20 | 20 | 20 | 20 | 20 | | 20 |
| *. Correlation is significant at the 0.05 level (2-tailed). | | | | | | | | | | | |  |  |
| **. Correlation is significant at the 0.01 level (2-tailed). | | | | | | | | | | | |  |  |

| **Supplementary Table 8.** Pearson correlation between paranormal beliefs and EEG bands power in left frontal lobe | | | | | | | | | | | |
| --- | --- | --- | --- | --- | --- | --- | --- | --- | --- | --- | --- |
|  | | 1 | 2 | 3 | 4 | 5 | 6 | 7 | 8 | 9 | 10 |
| 1.Paranormal beliefs | Pearson Correlation | 1 | -.380 | -.127 | -.455^*^ | -.316 | -.094 | -.356 | -.237 | .232 | -.276 |
|  | Sig. (2-tailed) |  | .098 | .593 | .044 | .175 | .693 | .123 | .314 | .324 | .239 |
|  | N | 20 | 20 | 20 | 20 | 20 | 20 | 20 | 20 | 20 | 20 |
| 2.Frontal_Lobe_total_Left | Pearson Correlation | -.380 | 1 | -.169 | .887^**^ | -.108 | -.237 | .754^**^ | .710^**^ | -.498^*^ | .781^**^ |
|  | Sig. (2-tailed) | .098 |  | .476 | .000 | .651 | .313 | .000 | .000 | .025 | .000 |
|  | N | 20 | 20 | 20 | 20 | 20 | 20 | 20 | 20 | 20 | 20 |
| 3.Frontal_Lobe_delta_Left | Pearson Correlation | -.127 | -.169 | 1 | -.012 | -.237 | -.218 | -.206 | -.018 | .221 | -.147 |
|  | Sig. (2-tailed) | .593 | .476 |  | .961 | .314 | .355 | .384 | .939 | .350 | .536 |
|  | N | 20 | 20 | 20 | 20 | 20 | 20 | 20 | 20 | 20 | 20 |
| 4.Frontal_Lobe_alpha_Left | Pearson Correlation | -.455^*^ | .887^**^ | -.012 | 1 | -.165 | -.316 | .675^**^ | .727^**^ | -.506^*^ | .650^**^ |
|  | Sig. (2-tailed) | .044 | .000 | .961 |  | .487 | .175 | .001 | .000 | .023 | .002 |
|  | N | 20 | 20 | 20 | 20 | 20 | 20 | 20 | 20 | 20 | 20 |
| 5.Frontal_Lobe_alpha1_Left | Pearson Correlation | -.316 | -.108 | -.237 | -.165 | 1 | .723^**^ | -.040 | -.254 | -.379 | -.065 |
|  | Sig. (2-tailed) | .175 | .651 | .314 | .487 |  | .000 | .867 | .281 | .100 | .787 |
|  | N | 20 | 20 | 20 | 20 | 20 | 20 | 20 | 20 | 20 | 20 |
| 6.Frontal_Lobe_alpha2_Left | Pearson Correlation | -.094 | -.237 | -.218 | -.316 | .723^**^ | 1 | -.075 | -.320 | -.239 | -.052 |
|  | Sig. (2-tailed) | .693 | .313 | .355 | .175 | .000 |  | .753 | .169 | .311 | .828 |
|  | N | 20 | 20 | 20 | 20 | 20 | 20 | 20 | 20 | 20 | 20 |
| 7.Frontal_Lobe_beta2_Left | Pearson Correlation | -.356 | .754^**^ | -.206 | .675^**^ | -.040 | -.075 | 1 | .887^**^ | -.479^*^ | .913^**^ |
|  | Sig. (2-tailed) | .123 | .000 | .384 | .001 | .867 | .753 |  | .000 | .032 | .000 |
|  | N | 20 | 20 | 20 | 20 | 20 | 20 | 20 | 20 | 20 | 20 |
| 8.Frontal_Lobe_beta1_Left | Pearson Correlation | -.237 | .710^**^ | -.018 | .727^**^ | -.254 | -.320 | .887^**^ | 1 | -.367 | .792^**^ |
|  | Sig. (2-tailed) | .314 | .000 | .939 | .000 | .281 | .169 | .000 |  | .111 | .000 |
|  | N | 20 | 20 | 20 | 20 | 20 | 20 | 20 | 20 | 20 | 20 |
| 9.Frontal_Lobe_beta_Left | Pearson Correlation | .232 | -.498^*^ | .221 | -.506^*^ | -.379 | -.239 | -.479^*^ | -.367 | 1 | -.402 |
|  | Sig. (2-tailed) | .324 | .025 | .350 | .023 | .100 | .311 | .032 | .111 |  | .079 |
|  | N | 20 | 20 | 20 | 20 | 20 | 20 | 20 | 20 | 20 | 20 |
| 10.Frontal_Lobe_gama_Left | Pearson Correlation | -.276 | .781^**^ | -.147 | .650^**^ | -.065 | -.052 | .913^**^ | .792^**^ | -.402 | 1 |
|  | Sig. (2-tailed) | .239 | .000 | .536 | .002 | .787 | .828 | .000 | .000 | .079 |  |
|  | N | 20 | 20 | 20 | 20 | 20 | 20 | 20 | 20 | 20 | 20 |
| *. Correlation is significant at the 0.05 level (2-tailed). | | | | | | | | | | | |
| **. Correlation is significant at the 0.01 level (2-tailed). | | | | | | | | | | | |

| **Supplementary Table 9.** Pearson correlation between paranormal beliefs and EEG bands power in left temporal | | | | | | | | | | | |
| --- | --- | --- | --- | --- | --- | --- | --- | --- | --- | --- | --- |
|  | | 1 | 2 | 3 | 4 | 5 | 6 | 7 | 8 | 9 | 10 |
| 1.Paranormal beliefs | Pearson Correlation | 1 | .013 | .033 | -.237 | -.248 | .360 | -.471^*^ | -.073 | .205 | -.315 |
|  | Sig. (2-tailed) |  | .958 | .889 | .315 | .292 | .119 | .036 | .760 | .385 | .176 |
|  | N | 20 | 20 | 20 | 20 | 20 | 20 | 20 | 20 | 20 | 20 |
| 2.Temporal_Lobe_delta_Left | Pearson Correlation | .013 | 1 | .038 | -.090 | -.232 | .053 | -.204 | -.144 | .579^**^ | -.424 |
|  | Sig. (2-tailed) | .958 |  | .874 | .707 | .324 | .824 | .387 | .544 | .007 | .062 |
|  | N | 20 | 20 | 20 | 20 | 20 | 20 | 20 | 20 | 20 | 20 |
| 3.Temporal_Lobe_theta_Left | Pearson Correlation | .033 | .038 | 1 | .587^**^ | -.310 | -.254 | .114 | .488^*^ | -.357 | .160 |
|  | Sig. (2-tailed) | .889 | .874 |  | .007 | .183 | .280 | .632 | .029 | .122 | .500 |
|  | N | 20 | 20 | 20 | 20 | 20 | 20 | 20 | 20 | 20 | 20 |
| 4.Temporal_Lobe_alpha_Left | Pearson Correlation | -.237 | -.090 | .587^**^ | 1 | -.244 | -.323 | .493^*^ | .732^**^ | -.570^**^ | .489^*^ |
|  | Sig. (2-tailed) | .315 | .707 | .007 |  | .299 | .165 | .027 | .000 | .009 | .029 |
|  | N | 20 | 20 | 20 | 20 | 20 | 20 | 20 | 20 | 20 | 20 |
| 5.Temporal_Lobe_alpha1_Left | Pearson Correlation | -.248 | -.232 | -.310 | -.244 | 1 | -.363 | .132 | -.177 | -.266 | .140 |
|  | Sig. (2-tailed) | .292 | .324 | .183 | .299 |  | .116 | .580 | .455 | .258 | .555 |
|  | N | 20 | 20 | 20 | 20 | 20 | 20 | 20 | 20 | 20 | 20 |
| 6.Temporal_Lobe_alpha2_Left | Pearson Correlation | .360 | .053 | -.254 | -.323 | -.363 | 1 | -.166 | -.241 | .404 | -.235 |
|  | Sig. (2-tailed) | .119 | .824 | .280 | .165 | .116 |  | .483 | .306 | .078 | .319 |
|  | N | 20 | 20 | 20 | 20 | 20 | 20 | 20 | 20 | 20 | 20 |
| 7.Temporal_Lobe_beta2_Left | Pearson Correlation | -.471^*^ | -.204 | .114 | .493^*^ | .132 | -.166 | 1 | .685^**^ | -.568^**^ | .907^**^ |
|  | Sig. (2-tailed) | .036 | .387 | .632 | .027 | .580 | .483 |  | .001 | .009 | .000 |
|  | N | 20 | 20 | 20 | 20 | 20 | 20 | 20 | 20 | 20 | 20 |
| 8.Temporal_Lobe_beta1_Left | Pearson Correlation | -.073 | -.144 | .488^*^ | .732^**^ | -.177 | -.241 | .685^**^ | 1 | -.676^**^ | .613^**^ |
|  | Sig. (2-tailed) | .760 | .544 | .029 | .000 | .455 | .306 | .001 |  | .001 | .004 |
|  | N | 20 | 20 | 20 | 20 | 20 | 20 | 20 | 20 | 20 | 20 |
| 9.Temporal_Lobe_beta_Left | Pearson Correlation | .205 | .579^**^ | -.357 | -.570^**^ | -.266 | .404 | -.568^**^ | -.676^**^ | 1 | -.616^**^ |
|  | Sig. (2-tailed) | .385 | .007 | .122 | .009 | .258 | .078 | .009 | .001 |  | .004 |
|  | N | 20 | 20 | 20 | 20 | 20 | 20 | 20 | 20 | 20 | 20 |
| 10.Temporal_Lobe_gama_Left | Pearson Correlation | -.315 | -.424 | .160 | .489^*^ | .140 | -.235 | .907^**^ | .613^**^ | -.616^**^ | 1 |
|  | Sig. (2-tailed) | .176 | .062 | .500 | .029 | .555 | .319 | .000 | .004 | .004 |  |
|  | N | 20 | 20 | 20 | 20 | 20 | 20 | 20 | 20 | 20 | 20 |
| *. Correlation is significant at the 0.05 level (2-tailed). | | | | | | | | | | | |
| **. Correlation is significant at the 0.01 level (2-tailed). | | | | | | | | | | | |

| **Supplementary Table 10.** Pearson correlation between paranormal beliefs and EEG bands power in right temporal | | | | | | | | | | | | |
| --- | --- | --- | --- | --- | --- | --- | --- | --- | --- | --- | --- | --- |
|  | | 1 | 2 | 3 | 4 | 5 | 6 | 7 | 8 | 9 | 10 | 11 |
| 1.Paranormal beliefs | Pearson Correlation | 1 | -.347 | -.080 | -.205 | -.357 | -.226 | .650^**^ | -.158 | -.369 | .287 | .040 |
|  | Sig. (2-tailed) |  | .133 | .737 | .385 | .122 | .338 | .002 | .506 | .109 | .220 | .868 |
|  | N | 20 | 20 | 20 | 20 | 20 | 20 | 20 | 20 | 20 | 20 | 20 |
| 2.Temporal_Lobe_total_Right | Pearson Correlation | -.347 | 1 | -.432 | -.263 | .427 | .511^*^ | -.219 | .588^**^ | .383 | -.600^**^ | .481^*^ |
|  | Sig. (2-tailed) | .133 |  | .057 | .263 | .061 | .021 | .354 | .006 | .096 | .005 | .032 |
|  | N | 20 | 20 | 20 | 20 | 20 | 20 | 20 | 20 | 20 | 20 | 20 |
| 3.Temporal_Lobe_delta_Right | Pearson Correlation | -.080 | -.432 | 1 | .613^**^ | .275 | -.370 | -.277 | .076 | .300 | .482^*^ | .044 |
|  | Sig. (2-tailed) | .737 | .057 |  | .004 | .240 | .108 | .237 | .750 | .198 | .031 | .854 |
|  | N | 20 | 20 | 20 | 20 | 20 | 20 | 20 | 20 | 20 | 20 | 20 |
| 4.Temporal_Lobe_theta_Right | Pearson Correlation | -.205 | -.263 | .613^**^ | 1 | .514^*^ | -.444^*^ | -.114 | -.066 | .440 | .146 | -.173 |
|  | Sig. (2-tailed) | .385 | .263 | .004 |  | .020 | .050 | .634 | .781 | .052 | .539 | .465 |
|  | N | 20 | 20 | 20 | 20 | 20 | 20 | 20 | 20 | 20 | 20 | 20 |
| 5.Temporal_Lobe_alpha_Right | Pearson Correlation | -.357 | .427 | .275 | .514^*^ | 1 | -.152 | -.179 | .514^*^ | .755^**^ | -.284 | .482^*^ |
|  | Sig. (2-tailed) | .122 | .061 | .240 | .020 |  | .522 | .450 | .020 | .000 | .226 | .031 |
|  | N | 20 | 20 | 20 | 20 | 20 | 20 | 20 | 20 | 20 | 20 | 20 |
| 6.Temporal_Lobe_alpha1_Right | Pearson Correlation | -.226 | .511^*^ | -.370 | -.444^*^ | -.152 | 1 | -.360 | .174 | -.037 | -.533^*^ | .087 |
|  | Sig. (2-tailed) | .338 | .021 | .108 | .050 | .522 |  | .119 | .464 | .877 | .016 | .714 |
|  | N | 20 | 20 | 20 | 20 | 20 | 20 | 20 | 20 | 20 | 20 | 20 |
| 7.Temporal_Lobe_alpha2_Right | Pearson Correlation | .650^**^ | -.219 | -.277 | -.114 | -.179 | -.360 | 1 | -.154 | -.160 | .285 | -.122 |
|  | Sig. (2-tailed) | .002 | .354 | .237 | .634 | .450 | .119 |  | .517 | .502 | .224 | .609 |
|  | N | 20 | 20 | 20 | 20 | 20 | 20 | 20 | 20 | 20 | 20 | 20 |
| 8.Temporal_Lobe_beta2_Right | Pearson Correlation | -.158 | .588^**^ | .076 | -.066 | .514^*^ | .174 | -.154 | 1 | .674^**^ | -.525^*^ | .861^**^ |
|  | Sig. (2-tailed) | .506 | .006 | .750 | .781 | .020 | .464 | .517 |  | .001 | .017 | .000 |
|  | N | 20 | 20 | 20 | 20 | 20 | 20 | 20 | 20 | 20 | 20 | 20 |
| 9.Temporal_Lobe_beta1_Right | Pearson Correlation | -.369 | .383 | .300 | .440 | .755^**^ | -.037 | -.160 | .674^**^ | 1 | -.359 | .392 |
|  | Sig. (2-tailed) | .109 | .096 | .198 | .052 | .000 | .877 | .502 | .001 |  | .120 | .087 |
|  | N | 20 | 20 | 20 | 20 | 20 | 20 | 20 | 20 | 20 | 20 | 20 |
| 10.Temporal_Lobe_beta_Right | Pearson Correlation | .287 | -.600^**^ | .482^*^ | .146 | -.284 | -.533^*^ | .285 | -.525^*^ | -.359 | 1 | -.417 |
|  | Sig. (2-tailed) | .220 | .005 | .031 | .539 | .226 | .016 | .224 | .017 | .120 |  | .067 |
|  | N | 20 | 20 | 20 | 20 | 20 | 20 | 20 | 20 | 20 | 20 | 20 |
| 11.Temporal_Lobe_gama_Right | Pearson Correlation | .040 | .481^*^ | .044 | -.173 | .482^*^ | .087 | -.122 | .861^**^ | .392 | -.417 | 1 |
|  | Sig. (2-tailed) | .868 | .032 | .854 | .465 | .031 | .714 | .609 | .000 | .087 | .067 |  |
|  | N | 20 | 20 | 20 | 20 | 20 | 20 | 20 | 20 | 20 | 20 | 20 |
| **. Correlation is significant at the 0.01 level (2-tailed). | | | | | | | | | | | | |
| *. Correlation is significant at the 0.05 level (2-tailed). | | | | | | | | | | | | |

| **Supplementary Table 11.** Pearson correlation between paranormal beliefs and EEG bands power in right parietal | | | | | | | | | | | |
| --- | --- | --- | --- | --- | --- | --- | --- | --- | --- | --- | --- |
|  | | 1 | 2 | 3 | 4 | 5 | 6 | 7 | 8 | 9 | 10 |
| 1.Paranormal beliefs | Pearson Correlation | 1 | .083 | -.005 | .085 | -.348 | -.244 | -.331 | -.378 | .222 | .013 |
|  | Sig. (2-tailed) |  | .729 | .985 | .721 | .133 | .300 | .155 | .100 | .347 | .957 |
|  | N | 20 | 20 | 20 | 20 | 20 | 20 | 20 | 20 | 20 | 20 |
| 2.Parietal_Lobe_delta_Right | Pearson Correlation | .083 | 1 | .612^**^ | .338 | -.352 | -.392 | -.117 | .029 | -.117 | .052 |
|  | Sig. (2-tailed) | .729 |  | .004 | .145 | .128 | .088 | .623 | .905 | .625 | .828 |
|  | N | 20 | 20 | 20 | 20 | 20 | 20 | 20 | 20 | 20 | 20 |
| 3.Parietal_Lobe_theta_Right | Pearson Correlation | -.005 | .612^**^ | 1 | .569^**^ | -.295 | -.429 | -.071 | .104 | .187 | .074 |
|  | Sig. (2-tailed) | .985 | .004 |  | .009 | .207 | .059 | .767 | .662 | .429 | .758 |
|  | N | 20 | 20 | 20 | 20 | 20 | 20 | 20 | 20 | 20 | 20 |
| 4.Parietal_Lobe_alpha_Right | Pearson Correlation | .085 | .338 | .569^**^ | 1 | -.428 | -.457^*^ | .234 | .496^*^ | -.035 | .077 |
|  | Sig. (2-tailed) | .721 | .145 | .009 |  | .060 | .043 | .321 | .026 | .885 | .746 |
|  | N | 20 | 20 | 20 | 20 | 20 | 20 | 20 | 20 | 20 | 20 |
| 5.Parietal_Lobe_alpha1_Right | Pearson Correlation | -.348 | -.352 | -.295 | -.428 | 1 | .957^**^ | .094 | -.057 | -.522^*^ | .007 |
|  | Sig. (2-tailed) | .133 | .128 | .207 | .060 |  | .000 | .692 | .810 | .018 | .977 |
|  | N | 20 | 20 | 20 | 20 | 20 | 20 | 20 | 20 | 20 | 20 |
| 6.Parietal_Lobe_alpha2_Right | Pearson Correlation | -.244 | -.392 | -.429 | -.457^*^ | .957^**^ | 1 | -.014 | -.171 | -.464^*^ | -.054 |
|  | Sig. (2-tailed) | .300 | .088 | .059 | .043 | .000 |  | .952 | .472 | .039 | .821 |
|  | N | 20 | 20 | 20 | 20 | 20 | 20 | 20 | 20 | 20 | 20 |
| 7.Parietal_Lobe_beta2_Right | Pearson Correlation | -.331 | -.117 | -.071 | .234 | .094 | -.014 | 1 | .902^**^ | -.511^*^ | .627^**^ |
|  | Sig. (2-tailed) | .155 | .623 | .767 | .321 | .692 | .952 |  | .000 | .021 | .003 |
|  | N | 20 | 20 | 20 | 20 | 20 | 20 | 20 | 20 | 20 | 20 |
| 8.Parietal_Lobe_beta1_Right | Pearson Correlation | -.378 | .029 | .104 | .496^*^ | -.057 | -.171 | .902^**^ | 1 | -.487^*^ | .369 |
|  | Sig. (2-tailed) | .100 | .905 | .662 | .026 | .810 | .472 | .000 |  | .029 | .109 |
|  | N | 20 | 20 | 20 | 20 | 20 | 20 | 20 | 20 | 20 | 20 |
| 9.Parietal_Lobe_beta_Right | Pearson Correlation | .222 | -.117 | .187 | -.035 | -.522^*^ | -.464^*^ | -.511^*^ | -.487^*^ | 1 | -.173 |
|  | Sig. (2-tailed) | .347 | .625 | .429 | .885 | .018 | .039 | .021 | .029 |  | .465 |
|  | N | 20 | 20 | 20 | 20 | 20 | 20 | 20 | 20 | 20 | 20 |
| 10Parietal_Lobe_gama_Right | Pearson Correlation | .013 | .052 | .074 | .077 | .007 | -.054 | .627^**^ | .369 | -.173 | 1 |
|  | Sig. (2-tailed) | .957 | .828 | .758 | .746 | .977 | .821 | .003 | .109 | .465 |  |
|  | N | 20 | 20 | 20 | 20 | 20 | 20 | 20 | 20 | 20 | 20 |
| **. Correlation is significant at the 0.01 level (2-tailed). | | | | | | | | | | | |
| *. Correlation is significant at the 0.05 level (2-tailed). | | | | | | | | | | | |

| **Supplementary Table 12.** Pearson correlation between paranormal beliefs and EEG bands power in left parietal | | | | | | | | | | | |
| --- | --- | --- | --- | --- | --- | --- | --- | --- | --- | --- | --- |
|  | | 1 | 2 | 3 | 4 | 5 | 6 | 7 | 8 | 9 | 10 |
| 1.Paranormal beliefs | Pearson Correlation | 1 | -.166 | -.276 | .059 | -.361 | -.136 | -.449^*^ | -.496^*^ | .239 | -.352 |
|  | Sig. (2-tailed) |  | .485 | .240 | .803 | .118 | .567 | .047 | .026 | .311 | .127 |
|  | N | 20 | 20 | 20 | 20 | 20 | 20 | 20 | 20 | 20 | 20 |
| 2.Parietal_Lobe_delta_Left | Pearson Correlation | -.166 | 1 | .412 | -.041 | -.104 | -.304 | .079 | .125 | -.142 | -.045 |
|  | Sig. (2-tailed) | .485 |  | .071 | .862 | .662 | .193 | .740 | .600 | .550 | .849 |
|  | N | 20 | 20 | 20 | 20 | 20 | 20 | 20 | 20 | 20 | 20 |
| 3.Parietal_Lobe_theta_Left | Pearson Correlation | -.276 | .412 | 1 | .529^*^ | -.106 | -.422 | .423 | .535^*^ | -.259 | .327 |
|  | Sig. (2-tailed) | .240 | .071 |  | .016 | .657 | .064 | .063 | .015 | .270 | .160 |
|  | N | 20 | 20 | 20 | 20 | 20 | 20 | 20 | 20 | 20 | 20 |
| 4.Parietal_Lobe_alpha_Left | Pearson Correlation | .059 | -.041 | .529^*^ | 1 | -.362 | -.348 | .240 | .416 | -.125 | .191 |
|  | Sig. (2-tailed) | .803 | .862 | .016 |  | .117 | .133 | .308 | .068 | .599 | .421 |
|  | N | 20 | 20 | 20 | 20 | 20 | 20 | 20 | 20 | 20 | 20 |
| 5.Parietal_Lobe_alpha1_Left | Pearson Correlation | -.361 | -.104 | -.106 | -.362 | 1 | .716^**^ | .175 | .026 | -.523^*^ | .162 |
|  | Sig. (2-tailed) | .118 | .662 | .657 | .117 |  | .000 | .461 | .913 | .018 | .496 |
|  | N | 20 | 20 | 20 | 20 | 20 | 20 | 20 | 20 | 20 | 20 |
| 6.Parietal_Lobe_alpha2_Left | Pearson Correlation | -.136 | -.304 | -.422 | -.348 | .716^**^ | 1 | -.121 | -.216 | -.375 | -.120 |
|  | Sig. (2-tailed) | .567 | .193 | .064 | .133 | .000 |  | .613 | .361 | .103 | .613 |
|  | N | 20 | 20 | 20 | 20 | 20 | 20 | 20 | 20 | 20 | 20 |
| 7.Parietal_Lobe_beta2_Left | Pearson Correlation | -.449^*^ | .079 | .423 | .240 | .175 | -.121 | 1 | .885^**^ | -.625^**^ | .918^**^ |
|  | Sig. (2-tailed) | .047 | .740 | .063 | .308 | .461 | .613 |  | .000 | .003 | .000 |
|  | N | 20 | 20 | 20 | 20 | 20 | 20 | 20 | 20 | 20 | 20 |
| 8.Parietal_Lobe_beta1_Left | Pearson Correlation | -.496^*^ | .125 | .535^*^ | .416 | .026 | -.216 | .885^**^ | 1 | -.556^*^ | .751^**^ |
|  | Sig. (2-tailed) | .026 | .600 | .015 | .068 | .913 | .361 | .000 |  | .011 | .000 |
|  | N | 20 | 20 | 20 | 20 | 20 | 20 | 20 | 20 | 20 | 20 |
| 9.Parietal_Lobe_beta_Left | Pearson Correlation | .239 | -.142 | -.259 | -.125 | -.523^*^ | -.375 | -.625^**^ | -.556^*^ | 1 | -.528^*^ |
|  | Sig. (2-tailed) | .311 | .550 | .270 | .599 | .018 | .103 | .003 | .011 |  | .017 |
|  | N | 20 | 20 | 20 | 20 | 20 | 20 | 20 | 20 | 20 | 20 |
| 10.Parietal_Lobe_gama_Left | Pearson Correlation | -.352 | -.045 | .327 | .191 | .162 | -.120 | .918^**^ | .751^**^ | -.528^*^ | 1 |
|  | Sig. (2-tailed) | .127 | .849 | .160 | .421 | .496 | .613 | .000 | .000 | .017 |  |
|  | N | 20 | 20 | 20 | 20 | 20 | 20 | 20 | 20 | 20 | 20 |
| *. Correlation is significant at the 0.05 level (2-tailed). | | | | | | | | | | | |
| **. Correlation is significant at the 0.01 level (2-tailed). | | | | | | | | | | | |

Supplementary Fig 1. The mediation model. The mediation model tests the relationship between inhibition (error in No-Go in Go/No-Go task) as a dependent variable, paranormal beliefs as an independent variable, frequency bands (delta, theta, alpha, alpha1, alpha2, beta, beta1, beta2, and gamma) as mediators. /: insignificant, *: significant in p>0.005.


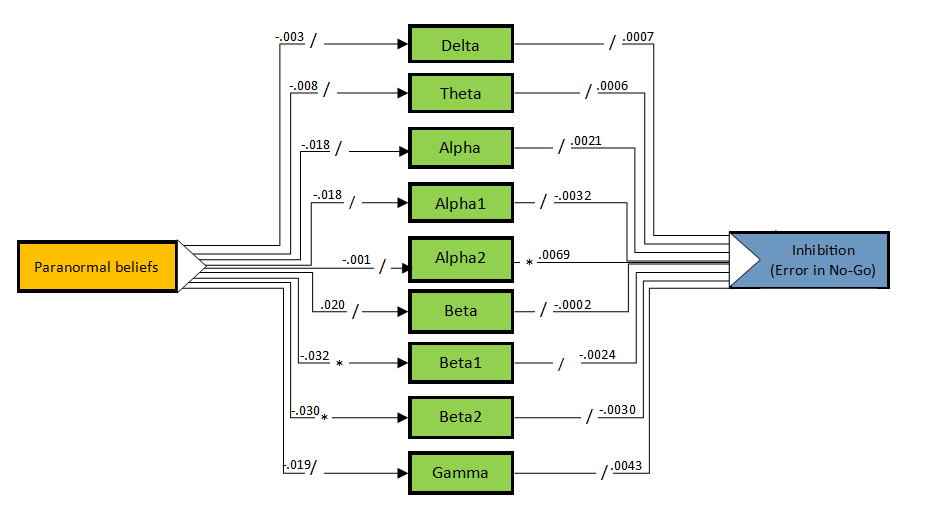

Supplement: Supplementary file 1 — Supplementary Information. [file 41598_2023_30457_MOESM1_ESM.docx]
